# The COP9 signalosome reduces neuroinflammation and attenuates ischemic neuronal stress in organotypic brain slice culture model

**DOI:** 10.1007/s00018-023-04911-8

**Published:** 2023-08-19

**Authors:** Yuan Tian, Jelena Milic, Laura Sebastián Monasor, Rahul Chakraborty, Sijia Wang, Yue Yuan, Yaw Asare, Christian Behrends, Sabina Tahirovic, Jürgen Bernhagen

**Affiliations:** 1 Vascular Biology, Institute for Stroke and Dementia Research (ISD), LMU Klinikum, Ludwig-Maximilian-University (LMU) Munich, Feodor-Lynen-Straße 17, 81377 Munich, Germany; 2 German Center for Neurodegenerative Diseases (DZNE), 81377 Munich, Germany; 3 Munich Cluster for Systems Neurology (SyNergy), Medical Faculty, LMU Munich, 81377 Munich, Germany; 4 Translational Stroke Research, Institute for Stroke and Dementia Research (ISD), LMU Klinikum, LMU Munich, 81377 Munich, Germany; 5 Present Address: Shenzhen People’s Hospital, Shenzhen, Guangdong Province China; 6 Present Address: Centre for Clinical Brain Sciences, The University of Edinburgh, Edinburgh, UK

**Keywords:** Constitutive photomorphogenesis 9 signalosome (CSN), CSN5, JAB1, Microglia, Ischemic stroke, Organoid

## Abstract

The constitutive photomorphogenesis 9 (COP9) signalosome (CSN) is a deNEDDylase controlling ubiquitination activity of cullin-RING-E3 ligases (CRLs) and thus the levels of key cellular proteins. While the CSN and its catalytic subunit CSN5 have been extensively studied in cancer, its role in inflammatory and neurological diseases is less understood. Following verification that CSN5 is expressed in mouse and human brain, here we studied the role of the CSN in neuroinflammation and ischemic neuronal damage employing models of relevant brain-resident cell types, an ex vivo organotypic brain slice culture model, and the CRL NEDDylation state-modifying drugs MLN4924 and CSN5i-3, which mimic and inhibit, respectively, CSN5 deNEDDylase activity. Untargeted mass spectrometry-based proteomics revealed that MLN4924 and CSN5i-3 substantially alter the microglial proteome, including inflammation-related proteins. Applying these drugs and mimicking microglial and endothelial inflammation as well as ischemic neuronal stress by TNF and oxygen-glucose-deprivation/reoxygenation (OGD/RO) treatment, respectively, we could link CSN5/CSN-mediated cullin deNEDDylation to reduction of microglial inflammation, attenuated cerebral endothelial inflammation, improved barrier integrity, as well as protection from ischemic stress-induced neuronal cell death. Specifically, MLN4924 reduced phagocytic activity, motility, and inflammatory cytokine expression of microglial cells, and this was linked to inhibition of inflammation-induced NF-κB and Akt signaling. Inversely, *Csn5* knockdown and CSN5i-3 increased NF-κB signaling. Moreover, MLN4924 abrogated TNF-induced NF-κB signaling in cerebral microvascular endothelial cells (hCMECs) and rescued hCMEC monolayers from OGD/RO-triggered barrier leakage, while CSN5i-3 exacerbated permeability. In an ex vivo organotypic brain slice model of ischemia/reperfusion stress, MLN4924 protected from neuronal death, while CSN5i-3 impaired neuronal survival. Neuronal damage was attributable to microglial activation and inflammatory cytokines, as indicated by microglial shape tracking and TNF-blocking experiments. Our results indicate a protective role of the CSN in neuroinflammation via brain-resident cell types involved in ischemic brain disease and implicate CSN activity-mimicking deNEDDylating drugs as potential therapeutics.

**Supplementary Information:**

The online version contains supplementary material available at 10.1007/s00018-023-04911-8.

## Introduction

Neuroinflammation is an underlying condition of various neurological diseases including ischemic stroke and Alzheimer’s disease [1–3]. The risk of stroke is increased by both acute and chronic inflammatory events. Both innate immune cells, in the acute ischemic phase, and adaptive immune cells, in the chronic phase, contribute to the neuroinflammatory status. Moreover, neuroinflammation plays a dual role in ischemic brain pathogenesis. The release of numerous inflammatory mediators promotes neuronal cell death by a variety of mechanisms. On the other hand, beneficial contributions come from anti-inflammatory signals that e.g. assist in post-ischemic wound repair [4–8]. Moreover, the initial inflammation that is triggered in the context of ischemia and necrotic neuronal cell death activates resident microglia and astrocytes, and promotes the infiltration of circulating peripheral leukocytes such as neutrophils, monocytes, and T cells through an impairment of blood–brain barrier integrity to aggravate inflammation, while also eliciting peripheral post-stroke immunosuppression [8, 9]. As brain-resident macrophage-like cells, microglia are first-line responders to ischemic injury. Their activation status has initially been described in terms of a binary classification as pro-inflammatory (M1-like) and anti-inflammatory/reparative (M2-like). M1-type microglia express pro-inflammatory mediators such as tumor necrosis factor-α (TNF-α), interleukin (IL)-1β, IL-6, and inducible nitric oxide synthase (iNOS), as well as matrix metalloproteinases (MMPs), which promote neuronal cell death and BBB disruption [10, 11]. Conversely, M2-type microglia are characterized by their production of IL-10, transforming growth factor-β (TGF-β), arginase-1 (Arg-1), and vascular endothelial growth factor (VEGF), which facilitate recovery following stroke [12]. However, this classification is over-simplified, as microglia appear in numerous overlapping states during ischemic stroke injury and neuroinflammation, as evidenced by recent single cell transcriptomic analyses, suggesting plasticity and a large heterogeneity and complexity [1, 13–15].

By controlling the expression of inflammatory cytokines, chemokines, and adhesion molecules, as well as regulating cell survival, the nuclear factor (NF)-κB pathway is a major signaling pathway driving microglial and neuroinflammation. Under homeostatic conditions, proinflammatory dimeric NF-κB transcription factors such as the p65/RelA-p50 heterodimer are kept in an inactive state in the cytosol by inhibitor of κB (IκB)-α [16–18]. Inflammatory stimulation via Toll-like receptors (TLRs) or TNF receptors leads to an activation of the IκB kinase (IKK) complex and phosphorylation of IκBα. Phosphorylated IκBα in turn is subjected to ubiquitination by the SCF-type cullin RING E3 ligase (CRL) SKP1-CUL1/RBX1-βTrCP (SCF^βTrCP^), followed by its degradation through the ubiquitin-26S proteasome (UPS) pathway. Ubiquitination of IκB thus releases the active NF-kB dimer, which translocates into the nucleus to trigger transcription of gene expression [17]. Numerous studies have shown that an attenuation of NF-kB activation prevents inflammatory gene expression and ameliorates neuroinflammation and ischemic stroke [19, 20].

Ubiquitination of IκBα by SCF^βTrCP^ is controlled by the constitutive photomorphogenesis 9 (COP9) signalosome (CSN), which thereby also controls inflammatory signaling through the NF-kB pathway [21]. The CSN is a conserved multiprotein complex of ~ 350 kDa, which was initially identified in *Arabidopsis thaliana* in 1992 and characterized as a light-regulatory locus, but was later on identified in all eukaryotes, including mammals [22–30]. The core CSN in mammals is composed of eight subunits designated CSN1 to CSN8, numbered according to their molecular size [31]. The crystal structure of the human CSN holo-complex was resolved at 3.8 Å resolution, confirming that the CSN has a striking architectural homology to the 19S lid sub-complex of the 26S proteasome and translation initiation factor 3 (elF3), and revealing the structural basis for CSN’s key function as an enzyme [32]. In fact, the CSN controls the activity of CRLs by removing the ubiquitin-like modifier neural precursor cell-expressed developmentally down-regulated 8 (NEDD8) from the cullin component of CRLs by a catalytic deNEDDylase activity [33, 34]. The actual catalytic deNEDDylase activity of the CSN is harbored within its subunit CSN5, formerly termed c-Jun activation domain binding protein-1 (JAB1). The CSN5 sequence comprises a JAB1/MPN/MOV34 (JAMM) Zn^2+^-metalloprotease motif that is embedded within the Mpr1, Pad1 N-terminal (MPN) domain of the protein, but only fulfils its catalytic action in the structural context of the CSN holo-complex [32]. The CSN also exhibits a deubiquitinase activity and promotes the deubiquitination of IκBα via the CSN-associated deubiquitinase USP15 [35]. Moreover, the CSN functions as an assembly platform for certain kinases, controlling additional key cellular functions beyond its role in regulating protein degradation via CRLs [36, 37].

Whereas the role of the CSN has been amply studied in cancer, its role in inflammatory diseases has only emerged relatively recently [38, 39]. When studying a conditional myeloid-specific knockout of *Csn5* in an atherogenic *Apoe*^*–/–*^ background, we previously uncovered a protective role of CSN5 (and the CSN) in atherosclerosis. This largely relied on the suppression of macrophage inflammation via deNEDDylation of CUL1, stabilization of IκBα and attenuation of NF-κB signaling. Moreover, applying MLN4924, a pharmacological inhibitor of the NEDD8-activating enzyme NAE1 thereby inhibiting the NEDDylation cascade, which mimics CSN5 hyperactivity and is used in clinical trials in patients with haematological and non-haematological malignancies [40–42], confirmed an anti-inflammatory activity of the CSN in macrophages, suggested a similar effect in inflammatory stimulated arterial endothelial cells, and led to partial atheroprotection in an *Apoe*^*–/–*^ mouse-based in vivo model of atherosclerosis [43]. Earlier work demonstrated a role for myeloid-expressed CSN5 in polymicrobial sepsis through deNEDDylation of CUL3 and control of MAP kinases [44]. CSN5 not only controls the expression and secretion of NF-κB-driven inflammatory mediators such as CCL2, but also is linked to the activity and secretion of the atypical inflammatory cytokine/chemokine MIF via the c-Jun kinase (JNK)/AP-1 pathway [45, 46]. The JNK/AP-1 axis also links CSN5 to inflammatory LFA-1 integrin activation [47]. Moreover, several other links between the CSN and cardiovascular disease have been uncovered [39].

In contrast, the role of CSN5 and the CSN in neuroinflammation is poorly understood and only a couple of reports are available. CSN3 was found to reduce neuroinflammation during cerebral ischemia/reperfusion injury through stabilizing suppressor of cytokine signaling 3 SOCS3 [48]. MLN4924 attenuated spinal cord ischemia–reperfusion (SCIR) injury in a rat model [49], and an early increase in mitochondrial localization of CSN5 was associated with larger stroke infarcts in female *Mif*-deficient mice [50]. Yu et al. have studied the acute phase after ischemic stroke in a mouse model and found that inhibition of NEDDylation by MLN4924 reduced infarct size and improved functional outcomes. Mechanistically, the protective effect of MLN4924 in the early acute stroke setting implicated the CRL substrate neurofibromatosis 1 (NF1) and a reduction in neutrophil infiltration [51].

Here, we hypothesized that the CSN could have a broader role in neuroinflammation and an important impact on brain-resident cell types that are activated and damaged during ischemic stress. We therefore studied its role in microglial homeostasis and inflammation, blood brain barrier (BBB) integrity, and neuronal death under ischemic conditions. We capitalized on single cell transcriptomic data base information, BV2 and primary murine microglia cells, mass spectrometry-based proteomics, microglial activation and signaling assays, cerebral microvascular endothelial cell cultures and BBB integrity assays, as well as mixed primary neuronal cultures and organotypic brain slices subjected to oxygen–glucose deprivation (OGD) stress. This was combined with approaches modulating the cullin NEDDylation status and CSN5 activity, i.e. by applying MLN4924 (which blocks cullin NEDDylation), the CSN5-specific small molecule inhibitor CSN5i-3 (which blocks CSN5 deNEDDylation activity), and CSN5 siRNA knockdown (which reduces the abundance of CSN5 protein and thus could also affect interactions between CSN5 and other proteins), respectively. Lastly, inflammation was blocked in OGD-stressed organotypic cultures by TNF-directed inhibitors. Our results are suggestive of a protective role of CSN5 and the CSN in neuroinflammation mediated by several cell types that are involved in ischemic brain disease.

## Materials and methods

### Chemicals, buffers, and miscellaneous reagents

MLN4924/Pevonedistat (product #A-1139) was bought from Active Biochem, China. A 500 μM stock solution was prepared in 100% DMSO. CSN5i-3 was obtained from MedChem Express (product #HY-112134). A 1 mM stock solution was prepared in 100% DMSO. Miscellaneous reagents were purchased from Sigma Aldrich/Merck (Darmstadt, Germany), VWR International GmbH (Darmstadt, Germany), Carl Roth GmbH (Karlsruhe, Germany), and ThermoFisher Scientific, Netherlands) and were of the highest purity degree available.

### Mice

Wildtype *C57BL/6J* mice were initially obtained from Charles River Laboratories (Sulzfeld, Germany). *Cx*_*3*_*cr1*^*EGFP/*+^ mice, which were originally obtained from the Jackson Laboratories (strain 005582; [52]), were established on a pure *C57BL/6 J* background. Mice were housed under standardized light–dark cycles in a temperature-controlled air-conditioned environment under specific pathogen-free conditions at the Center for Stroke and Dementia Research (CSD), Munich, Germany, with free access to food and water. Animals were sacrificed under anaesthesia with a mixture of midazolam (5 mg/mL), medetomidine and fentanyl (MMF). Mouse maintenance and experiments were reviewed and overseen by the institutional animal use and care committee of the local authorities (Regierung von Oberbayern, ROB, Germany) and performed in accordance with the procedures provided by the animal protection representative of CSD.

### Cell culture and cell lines

Cells were cultivated in a temperature- and humidity-controlled incubator at a temperature of 37 °C and 5% CO_2_. Fetal calf serum (FCS) from an EU-approved origin was obtained from Invitrogen-ThermoFisher Scientific (Karlsruhe, Germany) and heat-inactivated prior to usage. Other cell culture reagents, media and supplements were also bought from Invitrogen-ThermoFisher Scientific.

#### Cell culture of BV2 microglia

The BV2 microglia cell line CVCL_0182 was initially obtained from Dr. Markus Kipp (formerly RWTH Aachen University, Germany; now University of Rostock, Germany), who obtained the cells from Interlab Cell Line Collection (ICLC), Italy (accession number: ICLC ATL03001; http://wwwsql.iclc.it/det_list.php). The BV2 cell line was cultured in RPMI1640 (Roswell Park Memorial Institute 1640) GlutaMAX medium containing 10% FCS, 1% penicillin/streptomycin (P/S), and maintained in poly-l-ornithine (0.01%)-coated T75 flasks (Sigma Aldrich, Taufkirchen, Germany) in a humidified incubator at 5% CO_2_ and 37 °C. Cells were split every 2–3 days until passage 20. For inflammatory stimulation experiments, BV2 cells were stimulated with 20 ng/mL or 100 ng/mL mouse TNF-α (product # 300-01A, Peprotech, Hamburg, Germany) as indicated. For CSN5 mimicry or inhibition experiments, cells were pre-treated with 500 nM MLN4924 and 1 or 4 μM CSN5i-3, respectively, for 2–4 h in all experiments. Final DMSO concentrations in the vehicle groups were between 0.1% and 0.4% and did not interfere with cell viability as verified in scouting experiments.

#### Human cerebral microvascular endothelial cell culture (hCMEC/D3)

The immortalized hCMEC/D3 cell line was seeded on rat tail collagen type I (product #08115; Merck Millipore, Darmstadt, Germany) coated T-25 flask in EndoGRO-MV Complete Media Kit (product #SCME004; Merck Millipore), and maintained at 5% CO_2_ and 37 °C exposure. Cells were split when they reached a confluent monolayer. For stimulation experiments, hCMEC/D3 cells were treated with 100 ng/mL human TNF-α (product #300-01A, Peprotech) as indicated. Pre-treatment with MLN4924 and CSN5i-3 was performed as described above for BV2 cells (500 nM MLN4924 for 2 h, 1 μM CSN5i-3 for 4 h; final concentration of 0.1% and 0.1% DMSO in vehicle controls).

### Primary cell cultures

#### Primary neuronal culture

Primary neurons were derived from p0–p1 wildtype C57BL/6J neonatal mouse pups. Mice were decapitated by scissors and the heads immediately placed in ice-cold dissection buffer (97.5% HBSS Ca^2+^ and Mg^2+^ free, 110 μg/mL sodium pyruvate, 0.1% glucose, 10 mM HEPES, pH 7.3). Brain dissection was performed in the ice-cold dissection buffer in a 6 cm petri-dish under a Zeiss Stemi 305 microscope (Zeiss, Oberkochen, Germany). Cortical tissues were separated from the brain and the hippocampi removed. The cortex was transferred using a fire-polished glass Pasteur pipette into pre-warmed 2 mg/mL papain buffer at 37 °C for 15 min. The cell pellet was washed in cold plating medium (86.55% MEM Eagle’s with Earle’s BSS, 10% FCS, 0.45% glucose, 1 mM sodium pyruvate, 2 mM glutamine, 1% P/S once and triturated in pre-warmed plating medium using a pipette. After trituration, the total cell suspension was filtered through a 40 μm filter and cells plated on coverslips or dishes, coated with poly-l-Lysine (0.05 mg/mL). The plates were incubated at 37 °C in a humidified incubator with 5% CO_2_ and the medium replaced by growth medium (96% neurobasal medium, 2% B27, 2 mM glutamine, 1% P/S) the next day. Half of the growing medium was then changed twice a week for 10–14 days before any treatment. Neuronal cultures were pre-treated with MLN4924, CSN5i-3, or solvent control for two hours before exposure to OGD.

#### Primary microglia culture and mixed brain culture

Primary microglia were isolated from the cerebral cortex of p0–p2 wildtype C57BL/6J neonatal mouse pups and digested with papain following positive selection of CD11b + microglia/myeloid cells by CD11b magnetic bead enrichment (Miltenyi Biotec, Bergisch Gladbach, Germany). Isolated microglia were seeded in plates and grown in DMEM/F12 medium supplemented with 10% FCS, 1% P/S and 10 ng/mL GM-CSF. After 14 days, microglia were subjected to the indicated treatments.

CX_3_CR1^EGFP/+^ microglia migration was examined in mixed brain cultures. Brain cortices were isolated from CX_3_CR1^EGFP/+^ p0–p2 pups in Hank’s Balanced Salt Solution (HBS) supplemented with 8% NaHCO_3_ and 1 μM HEPES. Tissues were digested in papain solution and all cells plated in normal media (MEM without phenol red, 20% glucose, 8% NaHCO_3_, 0.1 mg/mL transferrin) with 0.5% P/S, 10% FCS, 2 mM l-glutamine, 0.025 mg/mL insulin in a 96-well cell imaging plate (product #0030741030; Eppendorf, Hamburg, Germany). Media were replaced on the second day by normal media and changed twice a week for 2 weeks. For inflammatory stimulation experiments of primary microglia cells/cultures, see “Cell culture of BV2 microglia” section.

### Transfection of BV2 microglia cells with siPool

BV2 cells were cultivated in the RPMI medium as described previously [53]. Specific siRNAs for the mouse *Cops5* gene were designed by siTOOL Biotech (Planegg, Germany). Transfection of BV2 cells was accomplished using Lipofectamine RNAiMax (product #13778075; Invitrogen-ThermoFisher Scientific) in Opti-MEM medium (product #31985070, Gibco-Invitrogen-ThermoFisher Scientific) according to the manufacturer’s protocol. Treatment of cells was started 48 h after transfection and was performed as indicated in Results.

### Proteomics analysis

BV2 cells were cultured in full medium in 10 cm cell culture dishes until they were confluent. MLN4924 (500 nM), CSN5i-3 (1 μM), or solvent control (0.01% DMSO) were added and the culturing continued for 6 h. After the incubation, cells were washed once with PBS, removed from the cell culture plate with a scraper, collected in tubes, and centrifuged in 1.5 mL Eppendorf tubes for 3 min to remove remaining PBS buffer, snap-frozen, and stored at −80 °C until further processing.

Thereafter cells were collected and lysed in urea buffer (9 M Urea, 50 mM Tris–HCl, pH 8.0, 150 mM NaCl, 1 × Roche protease inhibitor cocktail, product # 11836153001) followed by short sonification. Samples were cleared by centrifugation and protein amounts were adapted. Protein reduction was performed with dithiothreitol (DTT; 5 mM final) for 25 min at 56 °C and protein alkylation by the addition of iodoacetamide (14 mM final) for 30 min at room temperature. Protein mixtures were quenched with DTT and diluted 1:5 with 1 M Tris–HCl, pH 8.2. For increased peptide recovery, proteins were digested at room temperature for 3 h with LysC (FUJIFILM, 2 µL/100 µg protein) before overnight tryptic digest at 37 °C (0.5/100 µg protein). The following day, digestion was stopped with 10% trifluoroacetate (TFA). To increase analysis depth, samples were pre-fractioned by a C18-SCX custom-made stage tip [54]. Fractions were eluted stepwise with increasing NH_4_AcO concentrations (20–500 mM) and desalted on a separate C18 stage tip. Desalted peptides were loaded on a custom-made 75 mm × 15 cm fused silica capillary filled with C18-AQ resin (Reprosil Pur 120 HPLC column, 1.9 µm, Dr. Maisch HPLC GmbH, Ammerbuch, Germany) using an Easy-nLC1200 liquid chromatography. Samples were separated for 140 min with a 2.4–80% acetonitrile gradient in 0.1% formic acid using a Q Exactive HF mass spectrometer (MS; ThermoFisher Scientific). MS raw data were processed with MaxQuant (version 1.6.0.1) and loaded into Perseus (version 1.6.5.0), where matches to common contaminants, reverse identifications, identifications based only on site-specific modifications and with less than 2 peptides and MS/MS counts were removed. Only proteins with LFQ intensities in 3 out of 4 biological replicates in at least one experimental group were kept for the subsequent label-free quantification (LFQ). LFQ intensities were log_2_ transformed and missing values were replaced with random numbers drawn from a normal distribution. Student’s *t*-tests were used to determine the statistical significance of the abundance alterations of proteins detected between control and treated conditions. Proteins with *P* < 0.05, FDR < 0.05 and *t*-test difference > 0.5 or < −0.5 were considered to be significantly increased or decreased, respectively. Functional annotations were performed using DAVID (https://david.ncifcrf.gov/).

### RNA isolation and quantitative real-time PCR (RT-qPCR)

Total RNA was extracted from cells using TRIzol (product #15596018, ThermoFisher Scientific). RNA was reverse-transcribed into cDNA using ReverAid First Strand cDNA Synthesis Kit (product #K1622, ThermoFisher Scientific). Quantitative RT-PCR was performed using a RotorGene thermocycler (Qiagen Corbett, Hilden, Germany) and cDNA quantified with SYBR green. Primers used were: (i) mouse forward and reverse primers of -*Ccl2*, 5′-CATCCACGTGTTGGCTCA-3′, 5′-GATCATCTTGCTGGTGAATGAGT-3′: *Il12-β*, 5′-TTCTCACCGTGCACATCC-3′, 5′-GACCGGCACTGAGAGGAG-3′; *β-actin*, 5′-GGAGGGGGTTGAGGTGTT-3′, 5′-GTGTGCACTTTTATTGGTCTCAA-3′; *Tnf-α*, 5′-CATCTTCTCAAAATTCGAGTGACAA-3′, 5′-TGGGAGTAGACA AGGTACAACCC-3′; *iNos*, 5′-GGAGCCTTTAGACCTCAACAGA-3′, 5′-AAGGTGAGCTGAACGAGGAG-3′; (ii) human forward and reverse primers of—*GAPDH*, 5′-AAGGTGAAGGTCGGAGTCAA-3′, 5′-AATGAAGGGGTCATTGATGG-3′; *ICAM1*, 5′-CCTTCCTCACCGTGTACTGG-3′, 5′-AGCGTAGGGTAAGGTTCTTGC-3′; *VCAM1*, 5′-TGCACAGTGACTTGTGGACAT-3′, 5′-CCACTCATCTCGATTTCTGGA-3′; *E-selectin*, 5′-GAGTGCACATCTCAGGGACA-3′, 5′-ACTGCCAGGCTTGAACATTT-3′; *CCL2*, 5′-AGTCTCTGCCGCCCTTCT-3′, 5′-GTGACTGGGGCATTGATTG-3′, respectively. The cycle time (Ct) values were normalized with β-actin and evaluated by the ΔΔCt method.

### Western blotting

Cells were lysed in NuPAGE-LDS lysis buffer containing 125 mM DTT. Protein fractions were separated by 11% or 15% SDS-PAGE and blotted onto a nitrocellulose membrane. A 3% bovine serum albumin (BSA) solution was used for blocking and the following primary antibodies were applied for target protein detection: anti-NF-kB IkB-alpha (product #9242S, Cell Signaling Technologies, Frankfurt am Main, Germany), anti-NF-kB pI-kB-alpha (product #9246S, Cell Signaling), anti-cullin1 (product #sc-17775, Santa Cruz, Heidelberg, Germany), anti-NEDD8 (product #2745S, Cell Signaling), anti-VE-Cadherin (product #sc-9989, Santa Cruz), anti-occludin (product #71-1500, ThermoFisher Scientific), anti-Jab1 (product # sc-13157, Santa Cruz), anti-CSN8 (product #PW8290, Enzo Life Sciences GmbH, Lörrach, Germany), anti-Akt (product #9272S Cell Signaling), anti-p-Akt (product #9275 Cell Signaling), anti-claudin-5 (product #341600, ThermoFisher Scientific), anti-β-actin (product #sc-47778, Santa Cruz). As secondary antibodies, HRP-conjugated anti-mouse (product #ab6820, Abcam, UK) or anti-rabbit antibodies (product #BYT-ORB43514, Biozol, Eching, Germany) were used. Blots were developed with SuperSignal West Dura Extended Duration Substrate (product #34076 ThermoFisher Scientific) and visualized applying an Odyssey® Fc imager (LiCOR, Hamburg, Germany) and quantified by ImageJ-FIJI version 2.0.0.

### Phagocytosis assay

BV2 cells and primary microglia were cultured into 24-well plates on coverslips at a density of 5 × 10^4^ and 1 × 10^5^ cells per well, respectively. Cells were pre-treated with MLN4924 or vehicle control for 2 h in serum-free medium. Cells then were incubated with or without TNF-α containing 0.05% fluorescently-labeled latex beads (product #L3030, Sigma-Aldrich, Taufkirchen, Germany) for 6 or 24 h. After incubation, cells were washed five times with ice-cold PBS, followed by fixation using 4% PFA for 15 min in the dark, and permeabilized with 0.2% Triton X-100 for 10 min. The slides were mounted with the mounting medium containing DAPI (product #H1200-10, Vectashield, Eching, Germany), and the phagocytosis rate scored by fluorescence microscopy on a Leica Dmi8 microscope. Phagocytosing cells were defined as follows: phagocytosing cells (%) = number of cells that contained latex beads/number of total cells as scored by DAPI-positivity.

### Transwell permeability assay

For the Transwell permeability assay, hCMEC/D3 cells were seeded at a density of 2 × 10^5^ cells/well in 300 μL endothelial medium (ENdoGRO-MV Complete Culture Media Kit SCME004, Merck Millipore) on the membranes of the Transwell inserts (6.5 mm Transwell-COL collagen-coated 0.4 μm Pore PTFE membrane insert 3495, Corning (Merck). Following treatment with MLN4924, CSN5i-3, or vehicle and/or OGD/RO (see above), 5 µM Lucifer yellow was added to the upper chamber of the Transwell insert and the device incubated for 60 min (5% CO_2_, 37 °C). For quantification, 100 µL of medium from the lower chamber was transferred to a black 96-well polystyrene plate and the Lucifer yellow signal measured at 530 nm (excitation at 485 nm) with a fluorescence microplate reader (Perkin Elmer Enspire). The apparent permeability (*P*_app_) was calculated by the following equation: *P*_app_ = (d*Q*/d*t*)/(*A* x *C*), where d*Q*/d*t* is the amount of drug (here Lucifer yellow) transport within a given time period; *A* is the surface area of the insert; and *C* is the initial concentration of the drug in the upper chamber at time 0 h.

### Migration of microglia

Mixed brain cells were seeded in a 96-well cell imaging plate (product #0030741030, Eppendorf) in Hibernate-A medium (product #A1247501, ThermoFisher Scientific), pre-treated with MLN4924 or vehicle control and stimulated with or without CXCL12 at a concentration of 100 ng/mL. Cells were monitored under the FITC-channel of a fluorescence microscope (Leica Dmi8) every 5 min for 14 h applying the live-imaging modality. Tracking analysis of cells was performed using the plugin ‘Manual Tracking’ from the ImageJ software package (National Institutes of Health, NIH, Bethesda, USA). Migration was further analyzed with the ‘Chemotaxis and Migration Tool’ software from Leica to assess the accumulated distance as a parameter of migration response.

### Organotypic brain slice cultures

Organotypic brain slice cultures were prepared as described before [55, 56] with modifications. Brains were removed from p5–8 neonatal wildtype *C57BL/6J* or *Cx*_*3*_*cr1*^*EGFP/*+^ mice by decapitation according to animal handling laws. Hippocampi and neocortices were dissected and, as depicted in Fig. 8, 350 μm-thick sagittal sections were cut from the hippocampal and cortical tissue, using a Mcllwain tissue chopper (Model TC752, Mickle Laboratory Engineering Company, Goose Green, UK). Intact sections were carefully selected under a Zeiss Stemi 305 dissection microscope in dissection medium (MEM, product #32360026 (Gibco-Invitrogen-ThermoFisher Scientific), containing 1% P/S and 10 mM Tris HCl, pH 7.2). Slices were incubated in cold dissection medium for 30 min before plating, and two slices were plated onto a polytetrafluoroethylene (PTFE) membrane insert (0.4 μm, 30 mm diameter, PICMORG50 from Merck-Millipore). Slice culture medium containing 50% HEPES-buffered Minimum Essential Medium (MEM) (product #32360026), 25% heat-inactivated horse serum (product #26050088, Merck-Sigma), 25% HBSS (product #14025050) and 1 mM l-glutamine (product #25030081) (all but horse serum from Gibco-Invitrogen-ThermoFisher Scientific) was changed one day after initial seeding and subsequently twice a week. Treatment was applied directly to the slice culture medium. Stock solutions of MLN4924 or CSN5i-3 were prepared by dissolving compounds in 100% DMSO at a concentration of 500 μM and 1 mM. Slice cultures were pre-treated with 10 μM MLN4924 or CSN5i-3 for 24 h (final DMSO concentration 2% and 1%, respectively) before further being exposed to oxygen–glucose deprivation (OGD) stress (see below).

### Oxygen–glucose deprivation (OGD) and reoxygenation (RO)

Oxygen glucose deprivation (OGD) was performed on day 10–14 of the primary neuronal cultures, on fully confluent monolayers of hCMEC/D3 cells, and on 14-day-old organotypic brain slice cultures. Following prior culture in glucose-containing medium, the cell culture medium was changed to DMEM without glucose, glutamine, and phenol red (product #A1443001, Gibco-Invitrogen-ThermoFisher Scientific), and flushed with a gas-mix of 95% N_2_ and 5% CO_2_ for 10 min before placing the cells/organotypic slices into a humidified hypoxia chamber (Hypoxie Glove Box HGB-090-1, Toepffer Lab Systems, Adelberg, Germany; 95% N_2_, 5% CO_2_, 1% O_2_, 45% humidity, 37 °C) for indicated time intervals. OGD was terminated by returning the cells/organotypic slices to normoxic conditions with glucose-containing medium (RO, reoxygenation).

### Cell counting kit-8 (CCK-8) viability assay

Primary neuronal cells were seeded at the same density in 96-well plates and incubated with 100 µL growth medium for 10–14 days. The cells were then treated with MLN4924, CSN5i-3, or vehicle control, followed by OGD stress for various time intervals as indicated. Cell viability was determined after a 24 h re-oxygenation period using the CCK-8 kit (product #96992, Sigma-Aldrich). Briefly, to each well of the 96-well plate, 10 µL CCK-8 reagent was added to the medium and cells incubated for 2 h at 37 °C. The spectrophotometric absorbance of each well was determined using a multi-label microplate reader (Enspire, Perkin Elmer LAS GmbH, Rodgau, Germany) at a wavelength of 450 nm.

### Evaluation of cell damage in organotypic brain slice cultures

Propidium iodide (PI, product # 81845, Sigma-Aldrich) was used to label damaged cells in the organotypic slice cultures. After pre-treatment with MLN4924 or CSN5i-3 and OGD/RO, brain slices were incubated with 7 μM PI for 15 min at 37 °C. Afterwards, images were directly acquired using a Leica Dmi8 microscope (Leica Microsystems GmbH, Wetzlar, Germany) with a 4 × dry objective. Randomly-chosen PI-positive areas were manually quantified and normalized to the respective total tissue area, using Image J-Fiji (version 2.0.0). For the pharmacological blockade experiments with anti-TNF drugs, the investigator scoring the PI-positive areas was blinded for group assignments.

### Microglia morphology changes in brain slice cultures and in vivo

In order to investigate microglia phenotypes, brain slice cultures were prepared from *Cx*_*3*_*cr1*^*EGFP/*+^ mice. Following pre-treatment with MLN4924 or CSN5i-3 and OGD/RO (for details, see above), representative pictures from brain slices at a 14-day in vitro (DIV) period were acquired before and after fixation using a 4 × or 20 × dry objective (Leica Dmi8 fluorescence microscope, Leica Microsystems GmbH) or 40 × oil immersion objective (Zeiss AiryScan 880 confocal microscope).

### Immunofluorescence staining of cells and organotypic brain slices

BV2 microglial cells, primary neuronal cells and hCEMC/D3 cells were cultured on coverslips in 24-well plates. Following the above-described treatments, cells were fixed with 4% paraformaldehyde (PFA) for 15 min in the dark, permeabilized with 0.2% Triton X-100 for 10 min, blocked with SuperBlock solution (product #37515 ThermoFisher Scientific) for 1 h, and incubated with primary antibody overnight at 4 °C using the following antibodies: anti-NF-kB p65 (product #8242, Cell Signaling, 1:500), anti-beta-tubulin III (product #T8578, Sigma-Aldrich), anti-VE-cadherin (product #F8 sc-9989, Santa Cruz Biotechnology, 1:100). After three washes with PBS, slides were incubated with secondary antibody (goat anti-rabbit conjugated to Alexa Fluor 555, 1:1000 or goat anti-mouse conjugated to Alexa Fluor 647, 1:1000) for 1 h, counter-stained with DAPI, and coverslips finally fixed, and sealed on the slides. Images were acquired with a fluorescence microscope (Leica Dmi8) or a confocal microscope (Zeiss 880 AiryScan). For determining the proportion of nuclear p65 in BV2 and hCMEC/D3 cells, the fluorescent intensity of nuclear p65 was manually quantified from randomly chosen p65-positive areas, which overlapped with DAPI positivity, and was normalized to the respective total field area. Quantification was performed with Image J-Fiji (version 2.0.0). VE-cadherin positivity in hCMEC/D3 cells was determined accordingly and quantified via Image J-Fiji as well.

Brain slices were fixed in 4% PFA for 1 h at room temperature and permeabilized in PBS containing 0.5% Triton X-100 (PBS-T) for 30 min. Afterwards, slices were cut with the membrane from the insert using forceps and placed into a wet chamber, followed by blocking with PBS containing 0.5% Triton X-100 and 5% normal goat serum (product #ab7481, Abcam). To stain for neurons, slices were subsequently incubated with the primary antibody NeuN (product #26795-1-AP, Proteintech, UK) at 1:500 dilution in blocking solution overnight at room temperature. After three 10 min washes with PBS-T, slices were incubated with secondary antibody (goat anti-rabbit antibody conjugated to Alexa Fluor 647, 1:250, for 3 to 5 h at room temperature in the dark). After three 10 min washes with PBS-T, slices were circled with white filling paste and mounted with Fluoromount aqueous mounting medium (catalog #F4680, Sigma-Aldrich) and analyzed with a fluorescence microscope (Leica Dmi8). NeuN-positive areas were manually quantified and normalized to the respective total tissue area using Image J-Fiji (version 2.0.0).

### Statistical analysis

Statistical analysis was performed using GraphPad Prism Version 8 software. Unless stated otherwise, data are represented as means ± standard deviation (SD). After testing for normality by Shapiro–Wilk test, data were analyzed either by two-tailed Student’s *t*-test, Mann–Whitney *U* test, ordinary one-way ANOVA as appropriate, or two-way ANOVA. To account for multiple comparisons, either Dunnett’s or Bonferroni posthoc multiple comparisons tests were applied as appropriate. For information on the statistical analysis of the proteomics/mass spectrometry data, see the dedicated paragraph “Proteomics analysis”. Differences with *P* < 0.05 were considered to be statistically significant.

## Results

### CSN5 is robustly expressed in human and mouse brain

**Fig. 1:**
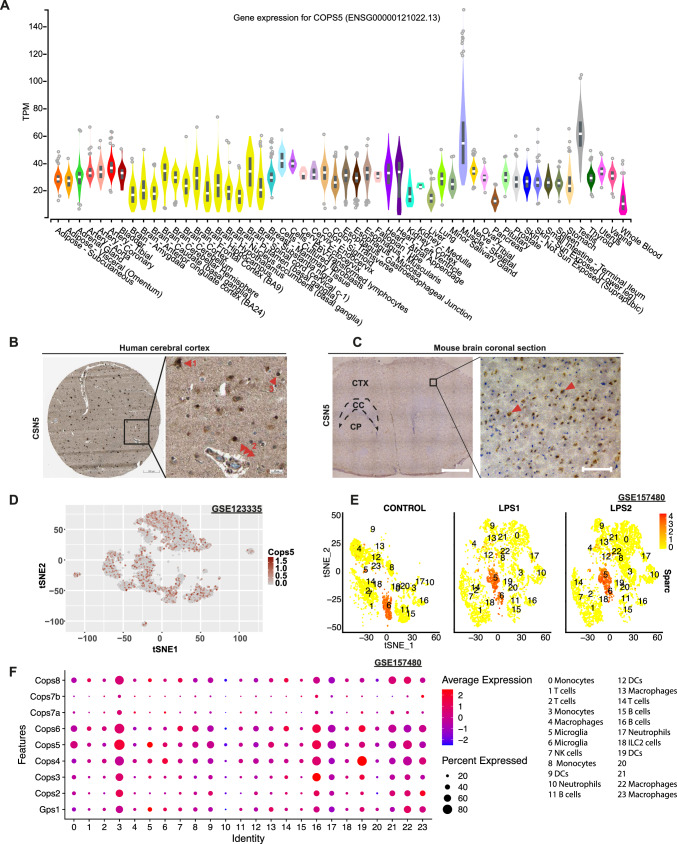
The COP9 signalosome and its subunit CSN5 are broadly expressed in the human and mouse brain. **A** Violin plots showing bulk gene expression profiles of CSN5 in healthy human tissues. Each color represents a type of tissue as indicated; TPM: transcripts per million. The figure was adapted from GTEx Analysis Release V8, https://www.gtexportal.org/home/gene/COPS5#$geneExpression). **B** Human cerebral cortex stained for CSN5. A neuronal cell (red arrow 1), glial cells (red arrow 3), and an area with typical histological features of a blood vessel (red arrow 2) showing positive staining for CSN5 are indicated. Scale bar: 200 µm (left), 25 µm (right) (credit: Human Protein Atlas; images obtained from https://www.proteinatlas.org/ENSG00000121022-COPS5/tissue and modified). **C** Representative image of an immunohistochemistry analysis of a brain section from C57BL/6J mice stained with a CSN5 antibody. Red arrows indicate CSN5-positive cells. Scale bar: 1 mm (left), 200 µm (right). *CTX* cortex; *CC* corpus callosum; *CP* caudoputamen. **D** Re-analysis of scRNAseq data from reference [101]. Feature plot visualizing expression levels of *Cops5* from clusters of cells (RNA-seq dataset: GSE123335). **E** Re-analysis of scRNAseq data from reference [76]. Feature plot visualizing expression levels of *Sparc* (microglial marker) from the clusters of immune cells (RNA-seq dataset: GSE157480) with the separated groups showing the cells from control or LPS-challenged brains. Red color intensity indicates the expression level of *Sparc* in each cell. **F** Dot plot visualizing *Cops1-8* expression levels and the percentage of cells within each cell cluster (average expression). Dots represent the degree of expression of each *Cops* subunit in all 23 identified clusters/cell types. Expression is illustrated both as relative average expression using color intensity from log + 2 to −2 (with red indicating high expression and blue indicating low expression), and as percent expression using size of the dots to indicate the percentage of Cops subunit-expressing cells within each cluster (with four categories shown: 20%, 40%, 60%, and 80%). DCs, dendritic cells; ILC2, type 2 innate lymphoid cells

The CSN is ubiquitously expressed, but the specific expression of its subunits on mRNA and/or protein level in the brain has not been systematically studied. To investigate the role of CSN5 and the other CSN subunits in neuroinflammation and ischemic brain pathologies, we first re-analyzed gene expression data from publicly available data repositories. Gene expression analysis of the human CSN5 gene *COPS5* from the GTEx Analysis Release V8 Data bank of healthy human tissue revealed that *COPS5* is prominently expressed in human brain, in particular frontal cortex and spinal cord, as well as in skeletal muscle, testis, artery, esophagus and other tissues, confirming ubiquitous expression of CSN5 and the CSN with a relatively low tissue specificity (Fig. 1A). The protein expression levels of CSN5 from the Human Protein Atlas confirmed high expression of CSN5 in cerebral cortex of human brain. This was also recapitulated by immunohistochemical analysis of mouse brain (Fig. 1B, C). Analysis of a recent single cell (sc) RNA-seq dataset (GSE123335) from healthy mouse brain demonstrated transcriptome-wide* COPS5* gene expression within all cell clusters including a cluster of microglia and other immune cells (Fig. 1D; Supplementary Fig. 1A). We next analyzed CSN5 and other CSN subunits in scRNA-seq data sets data sets of lipopolysaccharide (LPS)-challenged mice in sorted microglia and CD45^+^ non-microglia immune cells. Clustering of cell types (Fig. 1E; Supplementary Fig. 1B) in agreement with the whole brain scRNA-seq data set of Fig. 1D revealed that *Cops5* as well as the genes of the other Csn subunits (*Cops2, Cops3, Cops4, Cops6, Cops8, Cops7a, Cops7b*) were appreciably expressed in all immune cell types, while expression of e.g. *Cops4, Cops5, and Cops6* was highest in monocytes, B cells, macrophages, and dendritic cells. Levels overall did not substantially differ between healthy control brain and brain from LPS-challenged animals (Fig. 1F). We also re-analyzed an scRNA-seq data set from an experimental murine ischemic stroke study, in which mice undergoing middle cerebral artery occlusion (MCAO) were compared to sham-operated animals and single cell suspensions prepared from brain tissues at 24 h after MCAO and sham-operations (GSE174574). Csn subunit *Cops6* was most abundantly expressed and most Csn subunits, including *Cops5*, showed an upregulation upon MCAO challenge (Supplementary Fig. 2). Taken together, these data demonstrate that CSN5 is abundantly and broadly expressed in brain, including microglia and infiltrating immune cells, and is upregulated upon ischemic challenge, suggesting that it may have an essential role in brain homeostasis, ischemia, and neuroinflammation.

### Proteomic profiling reveals overlapping effects of abrogated cullin-1 NEDDylation and impaired CSN5 deNEDDylation activity on microglial pathways under basal culture stress

Owing to its potent inhibitory effect on the NEDDylation cascade, MLN4924 exhibits a CSN5-like anti-inflammatory activity [43]. Csn5i-3 binds to CSN5 while it resides in the CSN holocomplex and blocks its deNEDDylase activity, thus leading to an accumulation of NEDDylated cullins [57]. Here, we comprehensively studied the effect of these cullin NEDDylation state-modifying drugs in BV2 microglial cells, an immortalized murine cell line featuring many of the characteristics of primary microglia [53].

**Fig. 2:**
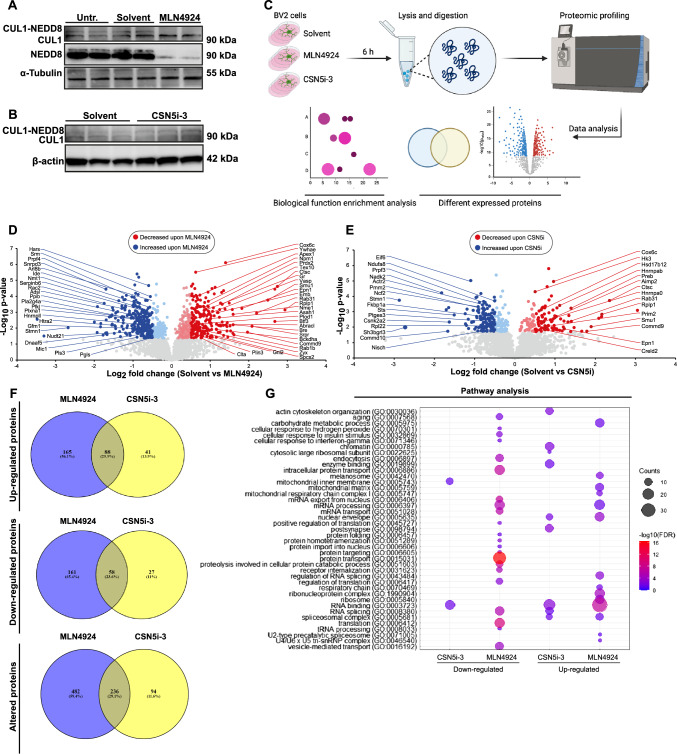
Proteomic profiling of differentially expressed proteins in BV2 cells treated with MLN4924 or CSN5i-3 versus solvent control. BV2 cells under basal conditions of culture stress were left untreated or were treated with control solvent (0.1% DMSO, termed solvent), MLN4924 (500 nM), or CSN5i-3 (1 μM) for 4 h before lysates for Western blot analysis were prepared, or treated for 6 h before cellular proteins were prepared for proteomic analysis. **A** Western blot analysis shows that MLN4924 inhibits CUL1 NEDDylation in BV2 cells. Upper panel, development of blot with an anti-CUL1 antibody; middle panel, development of blot with an anti-NEDD8 antibody; lower panel, α-tubulin as loading control. Each treatment was applied in duplicate. **B** Western blot analysis shows that CSN5i-3 increases CUL1 NEDDylation in BV2 cells. Upper panel, development of blot with an anti-CUL1 antibody; lower panel, β-actin as loading control. **C** Scheme depicting the experimental flow chart of the proteomics study. The scheme was created using Biorender (license ISD, LMU Munich). **D**, **E** Volcano plots using EnhancedVolcano package in R showing proteins with significant changes (−Log_10_ *q*) over differential expression between groups (log_2_ fold change). Red data points represent proteins exhibiting significative enrichment (*q* value < 0.05) and a log_2_ fold change > 1 between groups (solvent right, drug left). **D** Proteins differentially expressed between solvent- (right) and MLN4924- (left) treated BV2 cells. **E** Proteins differentially expressed between solvent- (right) and CSN5i-3- (left) treated BV2 cells. **F** Venn diagram showing overlapping proteins according to **C**–**E** shared between MLN4924 and CSN5i-3. Up- and down-regulated shared proteins and shared proteins among all altered proteins are shown as indicated. **G** Gene ontology (GO) term enrichment analysis of the significantly changed pathways derived from **D** and **E**, depicted as dot plot. Dots illustrate both the degree of significance, by which up- or down-regulation of a pathway was associated with MLN4924 or CSN5i-3 treatment, and the number of proteins populating a pathway. Counts, number of proteins associated with pathway; −log10(FDR), significance assigned to pathway

We first verified that MLN4924 blocks CUL1 NEDDylation in BV2 microglia similar to what we had previously observed in bone marrow-derived macrophages [43], and tested whether treatment of BV2 cells with CSN5i-3 led to an increase in the level of NEDDylated CUL1. Treatment of BV2 cells with MLN4924 for 4 h under basal conditions only entailing normal ‘cell culture stress’ led to a marked decrease in NEDDylated CUL1, accompanied by an increase in unmodified CUL1 levels, when compared to solvent control or untreated cells (Fig. 2A; Supplementary Fig. 3). This confirmed that MLN4924 blocks CUL1 NEDDylation in BV2. As expected, 4 h-treatment with CSN5i-3 showed an inverse effect. When compared with solvent control, CSN5i-3-treated BV2 cells showed a shift from the deNEDDylated cullin band to the NEDD8-CUL1 band (Fig. 2B).

To determine the effect of MLN4924 and CSN5i-3 on BV2 cell physiology under basal culture stress conditions, we performed comprehensive protein abundance profiling by mass spectrometry-based proteomics. Figure 2C illustrates the treatment scheme and proteomic work-flow and analysis by liquid chromatography tandem mass spectrometry (LC–MS/MS) of the generated peptides. Overall, 3878 proteins were detected and the number of detected proteins in the different treatment groups was comparable. Of these, 2158 proteins, which were detected in at least three out of the four replicates performed, were used for statistical analysis (Supplementary Table 1). 720 proteins were differentially regulated between the solvent and MLN4924 treatment groups, while 331 were significantly altered, when comparing the solvent group with CSN5i-3 (Supplementary Table 1).

The Volcano plot in Fig. 2D illustrates the proteins that were found differentially regulated when comparing the solvent- with the MLN4924-treated group. Proteins most highly enriched in solvent-treated BV2 cells encompassed proteins such as cathepsin C (Ctsc), the 60S acidic ribosomal protein P1 (Rplp1), the WD40 repeat-containing spliceosome protein Smu1, or cytochrome *c* oxidase subunit 6C (Cox6C). Highly enriched proteins in the MLN4924 treatment group were the cytoskeleton-regulatory proteins stathmin (Stmn1) and the Rho family GTPase Rac2, or the small nuclear ribonucleoprotein Sm D3 (Snrpd3). Additional markedly enriched proteins are annotated in Fig. 2D.

**Fig. 3:**
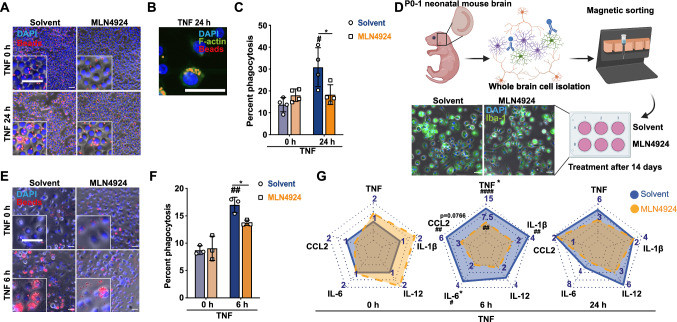
MLN4924 inhibits phagocytic activity and inflammatory cytokine production in inflamed microglial cells. **A**–**C** Phagocytosis of latex microbeads by BV2 microglia. **A** Representative images of BV2 cells pre-treated with solvent (left side) or MLN4924 (right side) followed by addition of red-fluorescent latex beads with or without TNF (20 ng/mL) stimulation for 24 h. Blue, DAPI-stained nuclei; red, latex beads. The insets show magnifications. Scale bar: 50 µm. **B** A representative magnified image showing latex microbeads phagocytosed by a BV2 cell following stimulation with TNF for 24 h (according to **A**, bottom left) is shown. Added latex microbeads are red-fluorescent. Cells were also stained for F-actin (green) and cell nuclei (DAPI, blue). Orange color indicates the overlay of endocytosed red latex microbeads colocalizing with green-fluorescent F-actin. Scale bar: 50 µm. **C** Quantification according to (**A**). The percentage of bead-positive cells was quantified (mean ± SD, *n* = 4 independent experiments; *P < 0.05, two-way ANOVA with Bonferroni post-test for comparing MLN4924 and solvent at 0 and 24 h TNF; ^#^*P* < 0.05, two-way ANOVA with Dunnett post-test for comparing TNF treated solvent control with unstimulated cells before adding TNF. **D**–**F** Phagocytosis of latex microbeads by sorted primary microglia from neonatal mouse brain. **D** Schematic representation of primary microglia isolation from C57BL/6J P0–1 pups using CD11b-based magnetic sorting. A representative magnified image showing isolated primary microglia as they were used for subsequent treatment/stimulation with solvent versus MLN4924. Scale bar: 50 μm. Immunofluorescence staining of Iba-1 (green) and DAPI (blue) in microglia left untreated (solvent) or treated with MLN4924 confirms microglia enrichment and cell integrity after isolation and solvent/MLN4924 pretreatment. The scheme was created using Biorender (license ISD, LMU Munich). **E** Representative images of primary microglia pretreated with solvent (left side) or MLN4924 (right side) followed by addition of red-fluorescent latex beads with or without TNF (20 ng/mL) for 6 h. Blue, DAPI-stained nuclei; red, latex beads. The insets show magnifications; scale bar: 50 μm. **F** Quantification according to (**E**). The percentage of bead-positive cells was quantified (mean ± SD, *n* = 3 independent experiments; *P < 0.05, two-way ANOVA with Bonferroni post-test for comparing MLN4924 and solvent at 0 and 24 h TNF; ^##^*P* < 0.01, two-way ANOVA with Dunnett post-test for comparing TNF-treated solvent control with unstimulated cells before adding TNF. **G** MLN4924 inhibits inflammatory cytokine expression in inflamed primary microglia. Spider/radar plot representation of proinflammatory gene expression at baseline (*left*) and 6 h (*middle*) or 24 h (*right*) of TNF stimulation, quantified by RT-qPCR (mean, *n *= 3–4; *P < 0.05, two-way ANOVA with Bonferroni post-test for comparison with solvent; ^#^*P* < 0.05, ^##^*P* < 0.01, ^####^*P* < 0.0001, two-way ANOVA with Dunnett post-test was performed for comparison with non-TNF-treated control in solvent or MLN4924 pre-treated group)

By inhibiting the NEDDylation of cullins, MLN4924 traps CRLs in an inactive state, preventing CRL substrate ubiquitylation and degradation. In contrast, treatment with CSN5i-3 blocks the deNEDDylase activity of the CSN and results in the accumulation of cullins in their NEDDylated, active, state. However, this seemingly opposite mechanism of regulating CRL NEDDylation may invoke similar cell-functional outcomes, since CSN5i-3-induced accumulation of hyper-active NEDDylated CRLs can lead to an auto-ubiquitination and degradation of substrate receptors by their CRLs. In turn, this results in reduced substrate ubiquitylation, similar to what is seen upon MLN4924 treatment [57]. Hence, when comparing solvent control with CSN5i-3 treatment (Fig. 2E), a similar differential profile was observed as for the comparison with MLN4924, with proteins such as Ctsc, Rplp1, or Smu1 highly enriched in the solvent group. When analyzing enriched proteins in the CSN5i-3 group, the degree of similarity appeared less pronounced, with proteins such as the redox-regulating proteins SH3 domain-binding glutamic acid-rich-like protein 3 (Sh3bgrl3) and NAD kinase 2 (Nadk2) found to be enriched in addition to Stmn1. Comparison of proteomic profiles of MLN4924- and CSN5i-3-treated BV2 cells revealed further potential similarities. The Venn diagrams in Fig. 3F demonstrate a substantial overlap of altered proteins between both drug treatments: (i) among the 294 proteins upregulated upon MLN4924 and CSN5i-3 treatment of BV2 cells, respectively, 30% or 88 proteins are shared between both groups (*P* = 6.19 × 10^–57^); (ii) among the 246 proteins down-regulated upon MLN4924 and CSN5i-3 treatment of BV2 cells, respectively, 24% or 58 proteins are shared (*P* = 1.64 × 10^–40^); (iii) among all 812 proteins altered upon MLN4924 and CSN5i-3 treatment of BV2 cells, respectively, 29% or 236 proteins are shared (*P* = 1.87 × 10^–54^). Thus, approximately one-third of all differentially regulated proteins are shared between both drug treatments. We also probed for potential overlaps of opposite effects of both drugs by Venn diagram analysis, but no shared proteins were found (Supplementary Fig. 4).

To more systematically unveil the signatures of differentially regulated proteins, we analyzed pathways associated with the altered proteins applying a gene ontology (GO) enrichment analysis. Pathways strongly down-regulated in the MLN4924 treatment group were related to: (i) protein transport and translation (intracellular protein transport, protein import into nucleus, protein targeting, folding, regulation of translation); (ii) endocytosis/vesicle-mediated transport (endocytosis, receptor internalization, vesicle-mediated transport, proteolysis); (iii) RNA processing and transport (mRNA export from nucleus, mRNA processing and transport); (iv) cell stress and inflammation (aging, cellular responses to hydrogen, insulin, and interferon-γ). Down-regulation of an RNA-processing pathway was also noted in the CSN5i-3 group. RNA biology-associated pathways were also prominently found in the group upregulated by MLN4924 treatment. However, in addition, this group featured pathways related to energy metabolism and mitochondria. Pathways upregulated by CSN5i-3 additionally encompassed terms related to actin cytoskeleton and chromatin. Pathways related to the terms RNA, spliceosome, and ribosome were also obtained, when using the Kyoto Encyclopedia of Genes and Genomes (KEGG) or InterPro databases (Supplementary Table 1).

Moreover, the identified terms in conjunction with key differentially expressed proteins such as Ctsc, Sh3bgrl3, Cox6C, or Stmn1 linked the microglial response to inflammation and metabolic/ischemic stress. Indeed, Volcano plot analysis with a focus on proteins related to the lysosome-endosome-phagocytosis-proteolysis pathway highlighted proteins such as Ctsc, the cysteine protease legumain, the endocytic accessory protein Epn1, or the vesicle-associated membrane protein 8 Vamp8, enriched in the solvent group (Supplementary Fig. 5A). Furthermore, proteins related to mitochondrial activity and the tricarboxylic acid cycle (TCA) such as interferon-regulated gene-1 (Irg1; also known as aconitate decarboxylase 1, Acod1) and isocitrate dehydrogenase [NAD] subunit gamma (Idh3g) were enriched, which play a role in controlling the levels of the immunometabolite itaconate [58] (Supplementary Fig. 5B).

### Inhibition of cullin-1 NEDDylation by MLN4924 reduces the microglial inflammatory response

The proteomics analysis indicated that, in addition to effects of MLN4924 and CSN5i-3 on basic cellular pathways, these drugs led to alterations in genes/pathways related to endocytosis/phagocytosis/vesicular trafficking, suggesting regulation of the microglial inflammatory response. We thus next assessed whether modulation of the CUL1 NEDDylation state has an effect on the activity of ‘inflamed’ microglia and used TNF as a surrogate mediator to trigger inflammation.

We first determined the phagocytotic activity of BV2 microglia in response to inflammatory stimulation. Indeed, microglial cells substantially enhanced their uptake activity for red-fluorescently labeled latex microbeads following stimulation with TNF (Fig. 3A). Magnification of the microscopic images confirmed intracellular localization of phagocytosed beads (Fig. 3A insets and B, the latter also showing colocalization of taken up beads with F-actin staining). Pre-treatment of BV2 cells with MLN4924 significantly inhibited TNF-stimulated uptake of latex microbeads (Fig. 3A, C). To confirm this effect of MLN4924, we next analyzed Iba1^+^ primary microglial cells isolated from P0–1 mouse brain pups by CD11b bead enrichment (Fig. 3D). Similar to the result obtained with BV2, stimulation with TNF enhanced the capacity of primary microglia to phagocytose latex beads, and MLN4924 blocked their phagocytotic activity (Fig. 3E, F).

Moreover, inhibition of CUL1 NEDDylation by MLN4924 in primary microglia led to a significant decrease in TNF-stimulated gene expression of *Il-6* and *Tnf* after 6 h of inflammatory stimulation with a trend of reduction observed for *Ccl2*, whereas the gene expression of *Il-12* and *Il-1*β was not significantly affected, as indicated by the spider plot in Fig. 3G. The attenuating effect on key inflammatory genes is in line with the observation that in the proteomics screen of culture-stressed BV2 cells several inflammation-related proteins, including osteopontin/Spp1, embigin/Emb, galectin-1/Lgals1, d-dopachrome tautomerase/Ddt, or macrophage mannose receptor 1/Mrc1, were found to be altered between the solvent and MLN4924 groups (Supplementary Table 1). Together, these results suggested that inhibition of CUL1 NEDDylation by MLN4924 in inflamed microglia was associated with the suppression of phagocytic activity and inflammatory cytokine production of microglia, thus interfering with contributions of microglia to the neuroinflammatory response.

### MLN4924 attenuates NF-κB signaling and reduces microglia motility via reduced AKT activity

**Fig. 4:**
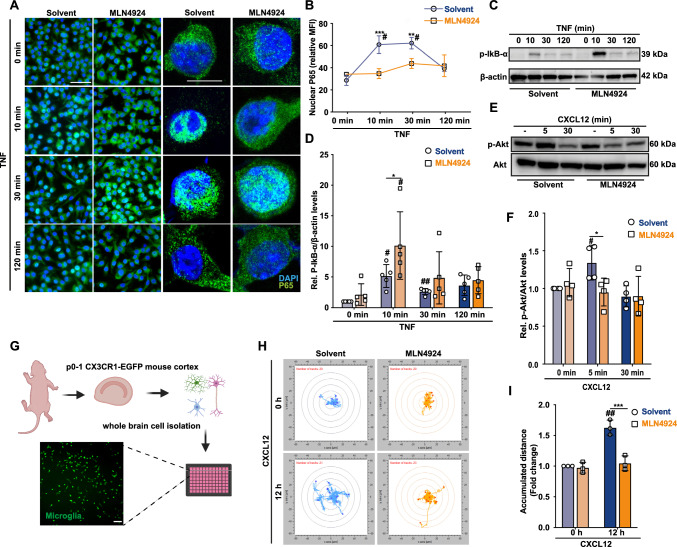
MLN4924 inhibits NF-κB and MAPK signaling in microglial cells and reduces microglia motility via suppressing the AKT pathway. **A**–**C** MLN4924 inhibits NF-κB signaling in BV2 microglia. BV2 cells pre-treated with solvent or MLN4924 and challenged with TNF-α for 0, 10, 30, and 120 min. **A** Immunofluorescent microscopy showing that MLN4924 blocked p65 nuclear translocation after 10 min of TNF-α induction. Translocation of p65 was visualized by using an Alexa Fluor 647-labeled anti-TNF-α antibody (green), and nuclei were counterstained by DAPI (blue). Representative images are shown. Scale bar: 50 µm (left) and 20 μm (right). **B** Quantification of nuclear P65 according to (**A**); mean ± SD of *n* = 3 independent experiments with 20 randomly chosen areas each; **P < 0.01, ***P < 0.001, two-way ANOVA with Bonferroni post-test for comparing the MLN4924-pre-treated group with the corresponding solvent group at the same time point; ^#^*P* < 0.05, two-way ANOVA with Dunnett post-test for comparing TNF-treated solvent control with the unstimulated group. MFI, mean fluorescent intensity. **C** Western blot analysis of p-IκB-α and β-actin levels in cell lysates from BV2 cells pre-treated with solvent or MLN4924 as challenged with TNF as indicated. **D** Quantification of (**C**). β-actin was used as loading control (mean ± SD, *n* = 5; *P < 0.05, two-way ANOVA with Bonferroni post-test for comparison with solvent; ^#^*P* < 0.05, ^##^P < 0.01, two-way ANOVA with Dunnett post-test for comparison of the solvent or MLN4924 pre-treated group with untreated control). **E**, **F** MLN4924 inhibits CXCL12-induced AKT phosphorylation in BV2 microglia. Cell lysates from BV2 cells pre-treated with solvent or MLN4924 and stimulated with the chemokine CXCL12 for different time intervals were analyzed for phosphorylated Akt (p-Akt) and total Akt by Western blot. **E** Representative Western blot. **F** Quantification according to (**E**) showing relative p-Akt/Akt ratios. Bars represent means ± SD, *n* = 4 biologically independent experiments; *P < 0.05, two-way ANOVA with Bonferroni post-test for comparison with solvent; ^#^*P* < 0.05, two-way ANOVA with Dunnett post-test for comparison of non-TNF/CXCL12-treated control with solvent or MLN4924-pre-treated group. **G**–**I** MLN4924 inhibits chemokine-elicited migration of Cx_3_cr1-EGFP + microglial cells in primary mouse mixed brain cell cultures. **G** Schematic representation of the preparation of mixed brain cell cultures from Cx_3_cr1-EGFP + p0–p1 mouse pups. A live fluorescence microscopic image showing Cx_3_cr1-EGFP + microglia in the mixed brain cell isolate after 14 days of culture is shown to illustrate enrichment of microglia. The scheme was created using Biorender (license ISD, LMU Munich). **H** Representative experiment showing microglia motility assessed by live-imaging of single-cell tracks, comparing MLN4924 with solvent control under both basal and CXCL12-stimulated conditions. Microglia pre-treated with solvent (blue) or MLN4924 (orange) followed by stimulation with (upper) or without (lower) CXCL12. **I** Quantification of (**H**); the accumulated distance of the tracks of 20–23 randomly selected cells per view, 4–5 views per treatment, three independent experiments, were recorded (mean ± SD; ***P < 0.001, two-way ANOVA with Bonferroni post-test was checked for comparison with solvent; ^##^*P* < 0.01, two-way ANOVA with Dunnett post-test was performed for comparison with non-CXCL12 treated control in solvent or MLN4924 pre-treated group)

Next, we investigated the underlying mechanisms by which blocking cullin1 NEDDylation reduces the inflammatory response in microglia. As a central mediator of pro-inflammatory gene induction, the NF-κB pathway is activated by TNF triggering phosphorylation of IκB-α via the IKK complex and its degradation via the UPS, a process controlled by NEDDylated SCF^βTrCP^ [17, 59, 60]. Therefore, we reasoned that MLN4924 would attenuate NF-κB signaling upon inflammatory stimulation in microglia. Our data show that MLN4924 delayed the onset of TNF-induced nuclear translocation of p65 from 10 to 30 min compared to control solvent (Fig. 4A, B). Consistently, MLN4924 markedly triggered the accumulation of phosphorylated IκB-α 10 min after TNF addition (Fig. 4C, D). Together, these results suggested that NEDDylated SCF^βTrCP^ promotes NF-κB signaling, thus enhancing the inflammatory response of microglia cells.

Along with NF-κB signaling, the phosphoinositide 3-kinase (PI3K)/Akt pathway also contributes to up-regulated cytokine production and inflammation in the central nervous system (CNS) [61]. We found that MLN4924 decreased phosphorylation of Akt in primary microglia stimulated with the chemokine CXCL12 (Fig. 4E, F). Interestingly, Akt signaling is a key pathway associated with microglia cell motility [62, 63]. We thus next studied the effect of MLN4924 on microglia migration in primary brain cultures isolated from *Cx*_*3*_*cr1*^*EGFP/*+^ mice, in which microglial cells are intrinsically labeled by EGFP (Fig. 4G–I). CXCL12-induced microglia motility, as recorded by total migrated distance of microglial cells, was significantly inhibited by MLN4924 co-treatment but not solvent control (Fig. 4H, I). These findings indicate that MLN4924 suppresses Akt signaling pathways in microglia, an effect accompanied by a reduction in microglia motility.

### CSN5 silencing and inhibition increases cullin1 NEDDylation and enhances NF-κB activity

**Fig. 5:**
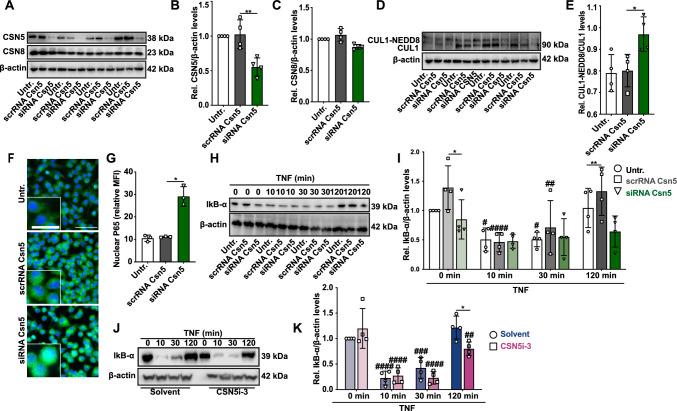
CSN5 silencing and its inhibition by CSN5i-3 increase CUL1 NEDDylation and activate the NF-κB pathway in microglial cells. **A**–**I** CSN5 silencing by siPOOL technology in BV2 microglia increases CUL1 NEDDylation and activates the NF-κB pathway. BV2 cells were transfected either with siPOOL which targets CSN5 (siRNA Csn5) or scrambled control (scrRNA Csn5) for 72 h; untr., untreated. **A** Western blot detection of CSN5 and CSN8 protein levels after siPOOL-mediated knockdown of Csn5. β-actin was used as loading control. **B** Densitometric quantification of Csn5 according to (**A**) expressed as ratio over β-actin and relative to untreated control, demonstrating a knockdown efficiency of approximately 50%. **C** Same as (**B**), except that densitometric quantification was performed for Csn8. **D** Western blot showing increased CUL1 NEDDylation by siPOOL-based knockdown of Csn5 in BV2 cells. β-actin was used as loading control. Four experiments were electrophoresed on the blot. **E** Bar graph showing quantification of (**D**) from four independent experiments (mean ± SD, *n* = 4; *P < 0.05, one-way ANOVA with Dunnett’s multiple comparison for comparison with scRNA control.). **F** Fluorescent microscopic images showing the nuclear localization of p65 in untreated BV2 cells or cells transfected with scrambled control or siPOOL against Csn5. Blue, DAPI-stained nuclei; green, p65 staining. Scale bar: 25 µm (inset, left) and 50 μm (right). A representative experiment is shown. **G** Quantification of nuclear p65 content according to (**F**); mean ± SD, *n* = 3 independent experiments; *P < 0.05, one-way ANOVA with Bonferroni post-test for comparing the siRNA CSN5 with the scrRNA group. **H, I** siPOOL-based knockdown of Csn5 leads to a decrease of IκB-α levels in TNF-stimulated BV2 cells. Western blot analysis of IκB-α levels in lysates from BV2 cells treated with TNF for the indicated time points; β-actin was used as normalization control. **H** Representative Western blot (four repeats). **I** Quantification of (**H**). Bars represent means ± SD, *n* = 4 independent experiments; *P < 0.05, **P < 0.01, two- way ANOVA with Dunnett post-test for comparison with scRNA control; ^#^*P* < 0.05, ^##^*P* < 0.01, ^####^*P* < 0.0001, two-way ANOVA with Dunnett post-test for comparison with non-TNF-stimulated control in untreated or scRNA/siRNA-treated group. **J**–**K** Inhibition of CSN5 deNEDDylation activity by CSN5i-3 in TNF-stimulated BV2 microglia activates the NF-κB pathway by attenuating IκB-α levels. For confirmation of an increasing effect on CUL1 NEDDylation see Fig. 2B. **J** Western blot analysis of IκB-α levels in BV2 cells after treatment with solvent *versus* CSN5i-3 and indicated intervals of TNF stimulation; a representative blot is shown. **K** Quantification of Western blots according to (**J**). Bars represent means ± SD, *n* = 4 independent experiments; *P < 0.05, two-way ANOVA with Dunnett post-test for comparison with solvent control; ^##^*P* < 0.01, ^###^*P* < 0.001, ^####^*P* < 0.0001, two-way ANOVA with Dunnett post-test for comparison with non-TNF-stimulated control in solvent or CSN5i-3-treated group

MLN4924 has been suggested to mimic CSN5 hyperactivation, but CSN-independent effects cannot be excluded, and potential off-target effects for MLN4924 have been described [64, 65]. To confirm the data obtained with MLN4924 and to gain more direct insight into the role of CSN5 in the neuroinflammatory response of microglia, we performed *Csn5* knock-down in BV2 microglia using siPOOL technology. The siPOOL silencing RNA cocktail directed against *Csn5* led to marked reduction in CSN5 levels of over 50%, both at mRNA (Supplementary Fig. 6) and protein (Fig. 5A, B) level. *Csn5* silencing did not lead to a significant reduction in Csn8 protein levels (Fig. 5C). Importantly, depletion of Csn5 was accompanied by an increase in CUL1 NEDDylation (Fig. 5D, E), implying that the partial depletion of *Csn5* was sufficient to reduce the functionality of the CSN holo-complex as a deNEDDylase.

Functionally, *Csn5* depletion led to an increased rate of TNF-triggered NF-κB p65 translocation from the cytoplasm into the nucleus (Fig. 5F, G) and to an attenuation of total IκB-α levels, with both basal IκB-α (before stimulation) and re-accumulated IκB-α levels 120 min after TNF stimulation decreased in the *Csn5* knockdown incubation, when compared with scrambled RNA control treatments (Fig. 5H, I), while phosphorylated IκB-α was not analyzed in this experimental setting.

We next wished to confirm the causal role of CSN5 on the microglia NF-κB signaling pathway by a pharmacological approach. Here, we capitalized again on the CSN5 inhibitor Csn5i-3 [57]. As shown before, Csn5i-3 led to enhanced NEDDylated CUL1 levels (Fig. 2B), verifying the principal efficiency of Csn5 inhibition by this compound in BV2 cells. When analyzing the functional effect of Csn5i-3 on NF-κB signaling, a similar outcome as that seen for genetic *Csn5* silencing was observed. Csn5i-3 led to a decrease in re-accumulated IκB-α levels 120 min upon TNF stimulation, although no effect on basal IκB-α levels was noted (Fig. 5J, K). Thus, the destabilizing effect of Csn5i-3 on IκB-α levels was inverse to the effect observed for MLN4924. This finding was in line with previous observations showing that the CRL substrate receptor βTRCP1 is not prone to undergo autoubiquitination and degradation upon CSN5 inhibition by Csn5i-3 [57]. Together, the genetic depletion of *Csn5* by siPOOLS silencing and the pharmacological blockade of Csn5 enzymatic activity by Csn5i-3, albeit entailing slightly different mechanisms of action (i.e. removal of Csn5, which may also affect protein–protein interactions of this subunit in addition to influencing the deNEDDylase activity, and inhibition of CSN5 protein residing in the holocomplex, respectively), showed an increase in NF-κB activity in BV2 microglia, mediated by increased IκB-α degradation, matching the inverse effects seen by MLN4924 treatment.

### MLN4924 suppresses NF-κB activation in inflammatory-elicited cerebral microvascular endothelial cells

Another cell type crucially involved in ischemic brain damage and the neuroinflammatory response are cerebral endothelial cells, key components of the blood–brain barrier (BBB) and integrity gate keepers. To determine how CSN5 affects BBB integrity and endothelial cell barrier functions, we treated hCMEC/D3 cells, primary human cerebral microvascular endothelial cells, whose stress activation and monolayer disruption is triggered by inflammatory factors including TNF [66], with MLN4924.

**Fig. 6:**
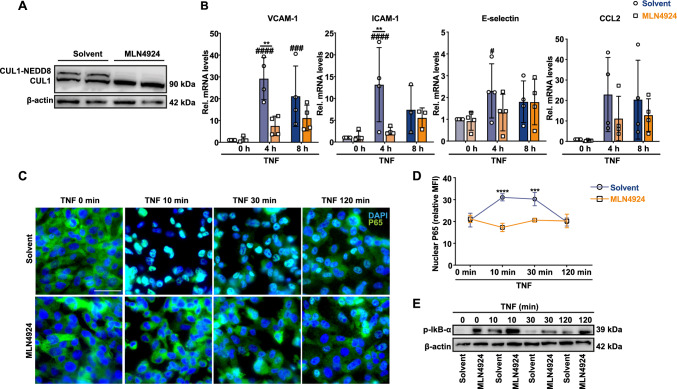
MLN4924 suppresses NF-κB activation in inflammatory-elicited microvascular cerebral endothelial cells. Human cerebral microvascular endothelial **(**hCMEC/D3) cells pretreated with solvent or MLN4924 were stimulated with or without TNF for the indicated time points. **A** Western blot developed with anti-CUL1 antibody shows that MLN4924 ablates CUL1 NEDDylation in hCMEC/D3 cells. **B** MLN4924 blocks TNF-induced upregulation of adhesion molecule expression. Relative *VCAM-1*, *ICAM-1*, *CCL2,* and *E-selectin* mRNA expression levels were measured by qRT-PCR in TNF-challenged hCMEC/D3 cells. mRNA levels were normalized to GAPDH and expressed relative to solvent without TNF treatment (mean ± SD, *n* = 3–4; **P < 0.01, two-way ANOVA with Bonferroni post-test for comparison with solvent control; ^#^*P* < 0.05, ^###^*P* < 0.001, ^####^*P* < 0.0001, two-way ANOVA with Dunnett post-test for comparison with non-TNF-treated control in solvent or MLN4924 pre-treated group). For E-selection and CCL2, only trends of induction and blockade, respectively, were noted. **C**–**E** MLN4924 reduces TNF-induced NF-κB activity in hCMEC/D3 cells. **C**, **D** NF-κB activation following TNF stimulation as indicated was determined by fluorescence microscopic detection of p65 nuclear translocation. **C** Representative images; blue, DAPI-stained nuclei; green, p65; size bar: 50 µm. **D** Quantification of nuclear p65 according to (**C**); mean ± SD of *n* = 3 independent experiments with 20 randomly chosen areas each; ***P < 0.001, ****P < 0.0001, two-way ANOVA with Bonferroni post-test for comparing the MLN4924-pre-treated group with the corresponding solvent group at the same time point; MFI, mean fluorescent intensity. **E** Immunodetection of p-IκB-α and β-actin in cell lysates of hCMEC/D3 cells following pre-incubation with MLN4924 and TNF stimulation as indicated. For an independent biological replicate of (**E**) see Supplementary Fig. 7

We first verified the effect of MLN4924 on CUL1 NEDDylation in these cells. In fact, MLN4924 led to an almost complete ablation of cullin NEDDylation (Fig. 6A), indicating interference with NAE-mediated NEDDylation. We next tested the effect of MLN4924 on the inflammatory response of these cells by determining adhesion molecule and chemokine expression. Treatment with TNF for 4 and 8 h markedly induced the gene expression of *VCAM-1* and *ICAM-1,* and MLN4924 blocked *VCAM-1* and *ICAM-1* upregulation after 4 h of TNF stimulation (Fig. 6B). E-selectin was also upregulated by TNF at the 4 h time point, but attenuation by MLN4924 did not reach statistical significance. For *CCL2,* neither the increase elicited by TNF nor the inhibitory effect of MLN4924 reached significance (Fig. 6B).

Capitalizing on our data obtained in microglia cells and based on the notion that adhesion molecule upregulation is chiefly driven by NF-κB, we next studied the effect of MLN4924 on NF-κB signaling. We first examined nuclear translocation of p65. MLN4924 blocked nuclear enrichment of p65 10 and 30 min after TNF stimulation, while the inhibitory effect vanished towards 120 min post TNF (Fig. 6C, D). Furthermore, MLN4924 led to a stabilization of phospho-IκB-α, both at baseline and at all time points following TNF stimulation, with the strongest effect seen at the 10 min stimulation interval (Fig. 6E; Supplementary Fig. 7). Collectively, these results suggested that MLN4924 inhibited CUL1 NEDDylation in cerebral microvascular endothelial cells and that this was accompanied by an attenuation of inflammation mediated via NF-κB signaling.

### MLN4924 reduces and CSN5i-3 exacerbates tight junction leakiness of cerebral microvascular endothelial cell monolayers induced by OGD/RO stress

**Fig. 7:**
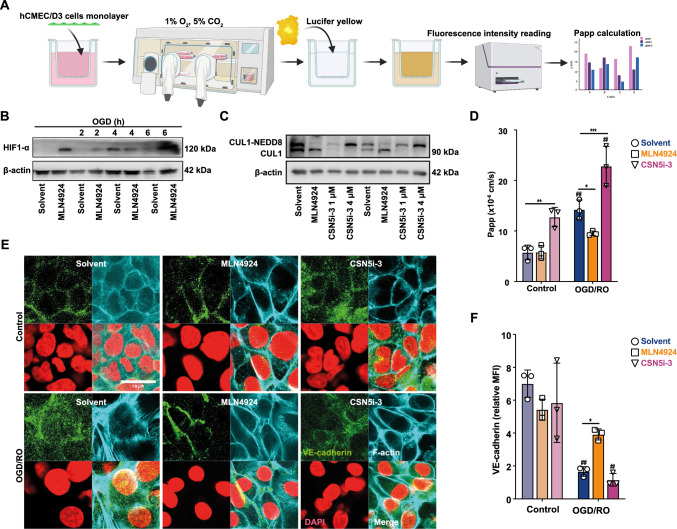
MLN4924 protects from and CSN5i-3 exacerbates barrier integrity loss induced by ischemia–reperfusion stress (OGD/RO) of microvascular cerebral endothelial cell monolayers. **A** Schematic representation of OGD/RO treatment of human cerebral microvascular endothelial (hCMEC/D3) cells and the Lucifer yellow-based assay for determination of endothelial barrier function. The scheme was created using Biorender (license ISD, LMU Munich). **B** Hypoxia (OGD) treatment induces HIF-1α and MLN4924 promotes HIF-1α stabilization. A representative Western blot out of two independent experiments developed with an antibody against HIF-1α is shown. β-actin was used as loading control. **C** MLN4924 blocks and CSN5i-3 promotes CUL1 NEDDylation in hCMEC cells. Western blot developed against CUL1 after duplicate treatments with solvent control, MLN4924 (500 nM), or the indicated concentrations of CSN5i-3. **D** MLN4924 protects from and CSN5i-3 exacerbates barrier leakage. Evaluation of hCMEC monolayer permeability determined by Lucifer yellow translocation and plotted as apparent permeability value Papp (mean ± SD, *n* = 3; *P < 0.05, **P < 0.01, ***P < 0.001, two-way ANOVA with Dunnett post-test for comparison with solvent; ^##^*P* < 0.01, two-way ANOVA with Bonferroni post-test for comparison with non-OGD/RO control in solvent, MLN4924 or CSN5i-3 pre-treated group). **E**, **F** MLN4924 preserves and CSN5i-3 impairs tight junction integrity of OGD/RO-stressed hCMECs. **E** Representative images showing fluorescence microscopic assessment of F-actin (cyan) and VE-cadherin (green) in solvent *versus* MLN4924- or CSN5i-3-treated hCMEC/D3 cells upon OGD/RO stress (*versus* control treatment) counterstained with DAPI (red). Scale bar: 20 μm. **F** Quantification of VE cadherin positivity at cell–cell contact areas according to (**E**); *MFI* mean fluorescent intensity

One of the hallmarks of ischemic brain stress and stroke is BBB dysfunction, allowing the infiltration of peripheral immune cells to exaggerate the inflammation [67]. Having established that CSN5 hyperactivity, as mimicked by MLN4924, attenuates inflammation in cerebral microvascular endothelial cells, we next examined the influence of NEDDylation/deNEDDylation on BBB function under conditions of ischemic stress, subjecting confluent hCMEC/D3 monolayers to oxygen–glucose deprivation (OGD) and reoxygenation (RO) stress (Fig. 7A). We first verified the experimental setting. Four hours of OGD led to a significant increase in HIF-1α levels, indicating effective hypoxic stimulation of hCMEC/D3 cells by the applied OGD protocol (Fig. 7B). MLN4924 was found to upregulate and stabilize HIF-1α, especially at baseline and after 2 h of OGD, when HIF-1α was otherwise not detectable in the solvent control group (Fig. 7B). The experiments in hCMEC cells also confirmed ablation of CUL1 NEDDylation by MLN4924 (and that an inverse effect was seen, when cells were treated with CSN5i-3, which led to an almost complete reduction of deNEDDylated CUL1 levels, demonstrating that this CSN5 inhibitor fully blocked the deNEDDylation activity of the CSN in hCMEC/D3 cells (Fig. 7C).

To directly determine the effect of CSN5 on BBB permeability, we used a Transwell set-up with hCMEC/D3 monolayers and Lucifer Yellow (LY) as a paracellular permeability marker detecting tight junction leakiness (see Fig. 7A). OGD/RO stress markedly enhanced permeability across confluent hCMEC/D3 monolayers, as indicated by an almost three-fold increase in the apparent permeability coefficient P_app_ for LY (Fig. 7D). MLN4924 protected against this loss of barrier integrity. CSN5i-3 exhibited an inverse effect and exacerbated the leakage, both under baseline conditions and in the context of OGD/OR stress (Fig. 7D). To explore the mechanism for this effect, we studied proteins involved in cell–cell contacts and tight junction formation by confocal fluorescence microscopy and Western blotting. Consistent with the above observations on leakiness, OGD/RO treatment led to a reduction and intracellular redistribution of vascular endothelial cadherin (VE-cadherin) levels with reduced VE-cadherin staining at cell–cell contact sites (Fig. 7E, F) and decreased overall VE-cadherin protein expression (Supplementary Fig. 8G). MLN4924 protected from these effects and reinstalled VE-cadherin levels and its distinct localization near cell surface regions, while CSN5i-3 exacerbated the adverse effect of OGD/RO treatment (Fig. 7E, F). Moreover, immunoblotting also indicated that the reduction in the levels of the tight junction proteins occludin and claudin-5 caused by OGD/RO was reversed by MLN4924, whereas CSN5i-3 had no effect (Supplementary Fig. 8). Together, these results support the view that CSN5 activity improves tight junction integrity of cerebral endothelial monolayers and reduces BBB permeability under OGD/RO stress.

### CSN5 activity attenuates ischemic neuronal damage in organotypic brain slice cultures: link to effect on microglial phenotype and inflammatory cytokines

**Fig. 8:**
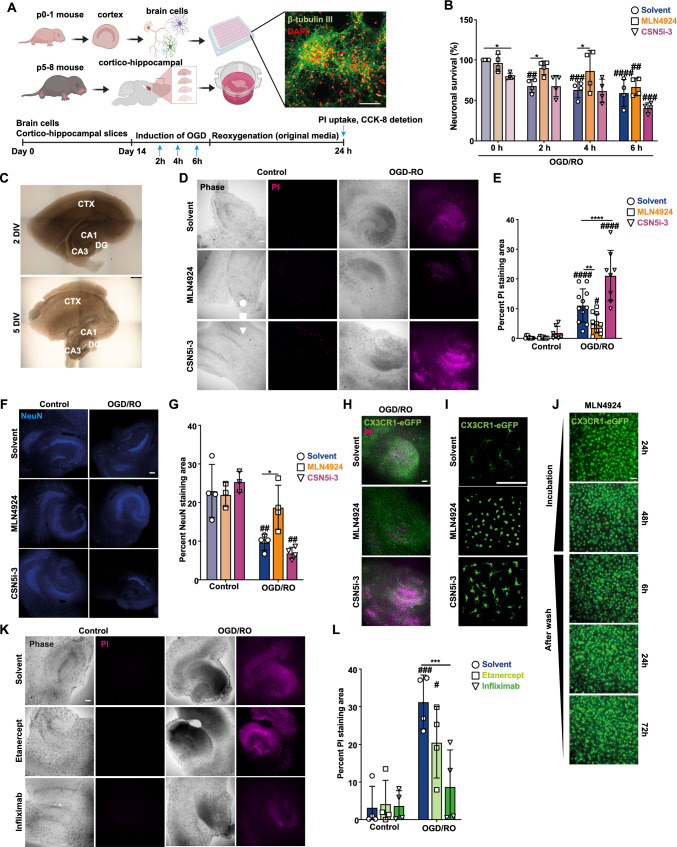
MLN4924 attenuates and CSN5i-3 aggravates neuronal damage in an ex vivo organotypic model of brain hypoxia/hyperoxia stress—role for microglial inflammation. **A** Cartoon summarizing both the experimental model of isolated primary neuronal cells and the organotypic cortico-hippocampal slice culture, both in the context of hypoxia/hyperoxia (oxygen–glucose deprivation/reoxygenation, OGD/RO) stress. The fluorescence staining of primary neurons positive for the neuronal marker β-III tubulin (green); counter-staining for DAPI-positive nuclei (red), illustrates successful enrichment of primary neuronal cells according to the flow shown in the upper panel. The scheme was created using Biorender (license ISD, LMU Munich). **B** MLN4924 promotes and CSN5i-3 impairs neuronal survival in primary murine neuronal cultures enriched according to **A**/upper panel. CCK8 assay showing neuronal cell viability of solvent, MLN4924, and CSN5i-3 pre-treated neuronal cells with or without OGD/RO challenge (mean ± SD, *n* = 4; *P < 0.05, two-way ANOVA with Dunnett post-test for comparison with solvent; ^##^*P* < 0.01, ^###^*P* < 0.001, ^####^*P* < 0.0001, two-way ANOVA with Dunnett post-test for comparison with non-OGD/RO control in solvent, MLN4924 or CSN5i-3 pre-treated group.) Each measurement was performed in triplicate, and at least five independent experiments were performed per treatment. **C**–**G** MLN4924 attenuates and CSN5i-3 aggravates neuronal damage in an ex vivo organotypic cortico-hippocampal slice culture model of brain hypoxia/hyperoxia (OGD/RO) stress. **C** Phase-contrast image of a control organotypic cortico-hippocampal slices after 2 and 5 days in vitro (DIV) maintenance. Scale bar: 500 μm;* CA* cornu ammonis; *CTX* cortex; *DG* dentate gyrus. **D** MLN4924 attenuates and CSN5i-3 aggravates OGD/RO-induced neuronal damage as assessed by PI staining of organotypic brain slice cultures. Representative images of PI uptake in slices treated with solvent, MLN4924, or CSN5i-3 and exposure to OGD/RO versus normoxic control treatment. Scale bar: 200 μm. **E** Quantification of the experiments according to (**D**). The area of PI-positivity (magenta) was normalized over total area. Bars represent mean ± SD, *n* = 6–9 independent experiments; **P < 0.01, ****P < 0.0001, two-way ANOVA with Dunnett post-test for comparison with solvent; ^#^*P* < 0.05, ^####^*P* < 0.0001, two-way ANOVA with Dunnett post-test for comparison with non-OGD/RO control in solvent, MLN4924, or CSN5i-3 pre-treated group. **F**, **G** Same as (**D**, **E**) except that neuronal viability was assessed by NeuN (blue) staining of brain slices normalized to the total area (**G**); means ± SD, *n* = 3–4; *P < 0.05, two-way ANOVA with Dunnett post-test for comparison with solvent; ^##^*P* < 0.01, two-way ANOVA with Bonferroni post-test for comparison with non-OGD/RO controls. (**H**–**J**) Effect of MLN4924 and CSN5i-3 on the morphology/shape of Cx3cr1-EGFP + microglia in organotypic brain slice cultures isolated from Cx3cr1-EGFP + pups. **H** Colocation of Cx3cr1-EGFP + microglia (green) and PI-positive cells (magenta) in brain slices exposed to OGD/RO stress. Representative images from five experiments are shown; scale bar: 200 μm. **I** Representative images of nine independent experiments with Cx3cr1-EGFP + microglia (green) showing different morphologies upon solvent, MLN4924, and CSN5i-3 exposure for 48 h. **J** Representative images of four independent experiments with Cx3cr1-EGFP + microglia morphology changing across the indicated time course of MLN4924 treatment and after wash-off. Scale bar: 50 μm. **K, L** Inhibition of TNF in OGD/RO-stressed organotypic brain slice cultures reduces neuronal damage. TNF inflammatory activity was blocked by incubation of slice cultures with the TNF-α-neutralizing drugs etanercept or infliximab. **K** Representative PI uptake images in slices treated with solvent, etanercept, or infliximab and exposed to OGD/RO stress or control conditions. Phase contrast images shown for comparison; scale bar: 200 μm. **L** Quantification according to (**K**). The PI-positive (magenta) area was normalized to the total area. Bars represent means ± SD, *n* = 4 biologically independent experiments; **P < 0.01, two-way ANOVA with Dunnett post-test for comparison with solvent; ^#^*P* < 0.05, ^###^*P* < 0.001, two-way ANOVA with Bonferroni post-test for comparison with non-OGD/RO controls

After establishing a beneficial effect of CSN5 and MLN4924 on microglia and cerebral endothelial cells, we finally studied their role on neuronal survival. This was first examined in β-tubulin III-positive primary neonatal neurons isolated from p0–1 mouse pups following exposure to OGD/RO stress (Fig. 8A, B). OGD/RO stress over a time course of 6 h significantly decreased neuronal viability in a time-dependent manner, with the lowest survival rate of approximately 60% seen at 6 h OGD/RO. Of note, MLN4924 was able to reverse OGD/RO-mediated neuronal death at the 2 and 4 h time intervals. In contrast, CSN5i-3 further reduced neuronal survival, although this effect only reached significance at the baseline time point (Fig. 8B).

To further examine the link between CSN5 and neuronal survival under OGD/RO stress, we applied organotypic brain slice cultures, suitable to mimic brain physiology/pathophysiology ex vivo [56, 68] (Fig. 8A, C). OGD/RO stress markedly increased propidium iodide (PI) uptake in such mouse cortico-hippocampal slice cultures (Fig. 8D, E), indicating substantially increased neuronal death. Of note, MLN4924 significantly protected from OGD-RO-induced death, whereas CSN5i-3 strongly enhanced PI uptake (Fig. 8D, E), a notion that was further supported by staining for the neuronal-specific marker NeuN, showing significantly increased staining in MLN4924-treated slice cultures (Fig. 8F, G). Notably, there was appreciable co-localization between CX3CR1-eGFP + microglia and the PI-positive cells in OGD/RO-stressed culture slices, suggesting that a presumed scavenger role of activated microglia is triggered by necrotic neuronal cells (Fig. 8H). This co-localization was reduced, when slice cultures were treated with MLN4924, whereas it was increased in CSN5i-3-treated slices (Fig. 8H). We also observed a morphology change of microglia following treatment with MLN4924 and CSN5i-3. Compared to solvent-treated OGD/RO-stressed slice cultures, CX3CR1-eGFP + microglia from MLN4924-treated OGD/RO-stressed cultures showed a pronounced amoeboid-like morphology, while CSN5i-3 exposure led to an intermediate phenotype (Fig. 8I) [69]. Microglia activation by OGD/RO treatment was confirmed by immunofluorescence staining for CD68, while the relative enhancement of CD68-positive microglia by CSN5i-3 at baseline conditions, was no longer seen after OGD/RO treatment (Supplementary Fig. 8). To further study the phenotype change and to determine if the alteration of microglia morphology by MLN4924 was reversible, we performed a kinetic experiment, in which MLN4924 was washed off after 48 h, and microglial cells followed for another 72 h. This showed that microglia continuously returned to a more ramified phenotype over the 72 h-recovery period (Fig. 8J).

Lastly, the ex vivo slice culture model was applied to determine whether the observed neuronal damage and microglia phenotype changes were dependent on locally produced inflammatory mediators. Because we had identified TNF as a key upregulated cytokine in inflammatory-stimulated primary microglia cultures that is blocked by MLN4924, we focused on a potential role of TNF in mediating neuronal damage and microglia activation in OGD/RO-treated brain slice cultures. OGD/RO-stressed slice cultures were co-treated with the neutralizing soluble TNF receptor fusion protein etanercept or the neutralizing anti-TNF antibody infliximab. Both drugs are applied in the clinic for autoimmune and inflammatory diseases [70]. PI uptake upon etanercept and infliximab was measured and compared to control solvent-treated cultures (Fig. 8K). The quantification revealed that infliximab led to a marked and significant reduction in PI + staining area, while the reduction seen for etanercept did not reach statistical significance (Fig. 8K, L). Together, this indicated that the brain inflammatory response associated with neuronal damage caused by OGD/RO stress in the ex vivo model is, at least partially, driven by TNF. In the context of our mechanistic data showing a protective role for CSN5 on TNF and NF-κB signaling in microglia and cerebral endothelial cell activation, we also conclude that protection by MLN4924/CSN5 hyperactivation from ischemic neuronal damage is partially mediated by attenuating the microglia—TNF-dependent—inflammatory response.

## Discussion

The CSN and, in particular, its catalytic subunit CSN5, plays an important role in cancer and its pro-tumorigenic mechanisms are well understood. In contrast, less is known about its role in inflammatory, cardiovascular and neurological diseases. In the present study we provide evidence for the role of CSN5 and the CSN in attenuating neuroinflammation and ischemic neuronal cell death. The evidence is based on in vitro experiments using microglial cells, cerebral endothelial monolayer cultures as a model for BBB integrity, neuronal cell cultures, as well as an ex vivo brain organoid model. Causality was studied by using the NEDDylation inhibitor MLN4924, CSN5i-3, an inhibitor of the catalytic deNEDDylation activity of CSN5, and by RNA silencing of Csn5. Using proteomics to study the effects of MLN4924 and CSN5i-3 on microglial cells under basal culture stress, we obtained a profile of how the CRL NEDDylation status shapes the microglial proteome. Inflammation as well as the ischemic neuronal stress response were mimicked by TNF and OGD/RO treatment challenges. We report on the first CRL NEDDylation state-dependent microglia proteome and link cullin deNEDDylation to reduction of microglial inflammation, attenuated cerebral endothelial inflammation, as well as protection from ischemia-induced neuronal cell death. While the CRL NEDDylation status broadly affected microglial pathways under basal stress, under inflammatory conditions, the microglial endocytosis/phagocytosis machinery, the NF-kB pathway, and AKT signaling, as well as TNF were identified as mediators counter-regulated by CSN/CSN5. While the CSN5 deNEDDylase activity is only operative in the structural context of the CSN complex, a limitation of our study is that we have not directly tested the role of other CSN subunits, e.g. by knockdown or knockout approaches. We thus cannot draw any firm conclusion about the involvement of the CSN holo-complex. In turn, it cannot be excluded that effects seen in the performed *Csn5* silencing experiments were, at least in part, mediated by complex-independent CSN5 activity.

The CSN is essential for cell physiology and its subunits are thus found ubiquitously expressed in all cell types and tissues. On the other hand, the aberrant overexpression of CSN subunits, most prominently CSN5 and CSN6, has been observed in human cancers including breast cancer [71], glioblastoma [72], and pancreatic carcinoma [73]. In contrast, the significance of altered CSN subunit expression levels in inflammatory, cardiovascular, or neurological diseases is incompletely understood [74, 75]. In the present paper, the expression levels and distribution of CSN subunits in mouse and human brain are reported for the first time. The data revealed that the expression of CSN5 (and its gene COPS5) in brain is prominent in neurons as well as microglia and is upregulated in experimental ischemic stroke, but seemingly not further upregulated upon inflammatory stimulation [76]. This is different from atherogenic inflammation [77] and inflamed cancer tissues [78, 79].

Disease-promoting mechanisms of the CSN in cancer affect the cell cycle and apoptosis [38, 80]. MLN4924 exerts anti-cancer effects [81, 82] by triggering apoptosis, senescence and autophagy [83]. CSN5i-3 suppresses tumor cell proliferation and growth of human xenografts in mice [57]. Given the opposite mechanistic effects of MLN4924 and CSN5i-3 on the NEDDylation status of CRLs (MLN4924: inhibition of cullin NEDDylation and CRL ubiquitin ligase activity; CSN5i-3: accumulation of cullins in their NEDDylated, active state), the effectiveness of both drugs on cancer cell behavior is at first sight surprising, but can be explained by the different susceptibility of various F box proteins/substrate receptor modules (SRMs) to CRL auto-ubiquitination activity [57]. Less is known about the effect of these CRL NEDDylation state-modifying drugs in inflammatory diseases. We previously showed that MLN4924 abrogated inflammatory cytokine expression in atherogenic cell types and attenuated atherosclerosis [43]. Furthermore, CSN8-mediated mechanisms play a role in the context of misfolded cardiac proteins and survival of cardiomyocytes in cardiomyopathies [84, 85]. Only a few studies have linked the CSN to the neuroinflammatory response and stroke: Liang et al. provided evidence that CSN3 stabilized SOCS3, an effect that leads to a restriction of the neuroinflammatory response [48]. Yu et al. showed that inhibiting NEDDylation with MLN4924 during the acute phase following ischemic stroke led to a reduction in infarct size and improvement in functional outcomes by reducing the infiltration of neutrophils. This beneficial effect was attributed to the accumulation of the CRL substrate NF1 [51]. However, the role of CSN5 in microglial biology, microglia-associated neuroinflammation, cerebral endothelial cell tight junction control, and the link of these mechanisms to ischemia-driven neuronal damage is poorly understood. In this regard, our study fills a mechanistic gap as to how CSN/CSN5 links to neuroinflammation and ischemic brain damage.

We first explored a potential influence of different CSN-driven CRL NEDDylation states on microglial physiology/activation under basal conditions of culture stress by determining mass spectrometry-based proteomic profiles of MLN4924- and CSN5i-3-treated BV2 microglial cells. Several observations were made: (i) MLN4924 and CSN5i-3 led to alterations in approximately 600 proteins with a roughly equal distribution of up- and down-regulated proteins; (ii) 30% of all altered proteins were shared for both drug treatments. At first sight, this observation appears to be counter-intuitive, as MLN4924 and CSN5i-3 affect the CRL NEDDylation status oppositionally. However, as discussed above similar functional outcomes can occur when CSN5i-3-induced accumulation of NEDDylated CRLs leads to auto-ubiquitination and degradation of certain SRMs [57]. (iii) Major altered pathways were protein transport and translation, endocytosis/vesicle-mediated transport, RNA processing and transport, as well as cell stress and inflammation. This was also reflected by the identity of the enriched proteins. Examples are Rab31 and Ctsc, which were found down-regulated by both MLN4924 and CSN5i-3. Shared altered inflammatory and immunometabolic proteins were for example legumain, Epn1, Irg1/Acod1, Idh3g, or Cox6c. In fact, a number of recent studies implicated knockouts of the asparagine endopeptidase legumain in improved cognitive impairment and neuroprotection, in the context MCAO [86]. Epn1 is an endocytic accessory protein that plays an important role clathrin-mediated endocytosis and has been suggested to control endocytotic sorting processes in cardiovascular diseases [87]. Irg1/Acod1, Idh3g, and Cox6c are key enzymes in mitochondrial energy production; the balance of catalytic activities of Irg1/Acod1 and Idh3g controls the levels of the immunometabolite itaconate. Itaconate, derived from cis-aconitate by Acod1 (encoded by *Irg1*) catalysis, is a key immune regulator implicated in macrophage inflammation and immunity, which acts by controlling succinate levels, glycolysis, and the NLRP3 inflammasome [58]. Itaconate has been suggested to display anti-inflammatory effects in several disease models, including ischemia/reperfusion injury [58].

We next assessed altered CRL NEDDylation states in microglial cells in the context of inflammatory stimulation, i.e. upon exposure to the inflammatory cytokine TNF. In line with our previous study in macrophages, inflammatory cytokine expression levels was upregulated in TNF-stimulated microglia and MLN4924 reduced the levels of *Tnf-α* and *Il-6*, indicating that it attenuates microglial inflammation [43]. Another characteristic similarity shared between microglia and macrophages is their ability to phagocytose, in turn a hallmark of the inflammatory response. Our observation that MLN4924 reduced TNF-induced microglia phagocytic activity (together with its observed effect on neuronal death upon OGD/RO) underscores a potential therapeutic value in conditions involving neuroinflammation and ischemic damage. Interestingly, SCF-dependent ubiquitylation and proteasomal degradation processes have been show to mediate phagocytosis [88].

Since the first observation that the expression of RelA/p65 and p50 was enhanced in human cerebral infarction [89], NF-κB has been considered a central modulator of neuroinflammation and stroke. For example, IKK/NF-κB-dependent microglia activation elevates kainic acid-induced neuronal death through induction of inflammatory mediators [90]. Moreover, previous studies described the involvement of the CSN in NF-κB activation in endothelial cells, myeloid cells, T cells, Hela cells, cardiomyocytes, cardiac fibroblasts, and MLN4924 was found to reduce NF-κB signaling in B cells, myeloid leukemia cells, cervical cancer cells, and macrophages [39]. In accord, we found that MLN4924 led to a rapid accumulation of IκB-α levels in microglial cells, whereas RelA/p65 nuclear translocation was inhibited. This notion was confirmed by experiments applying RNA silencing of *Csn5* and using CSN5i-3, which exhibited inverse effects on IκB-α and nuclear p65. This finding appears to be at odds with our observation in the proteomics screen, which showed that > 30% of identified proteins were shared between the MLN4924 and CSN5i-3 treatment groups. The explanation comes from the aforementioned differential susceptibility of certain CRL SRMs to undergo autoubiquitination and degradation upon CSN5 inhibition by Csn5i-3. In fact, the IκB-α-binding SRM βTRCP1 is not auto-ubiquitinated and degraded upon CSN5 inhibition by CSN5i-3 [57]. This explains the opposite directionality of effects of both inhibitors. Since a major part of the microglial inflammatory response measured in our study, as well as the barrier phenotype observed in the cerebral endothelial cell model, is driven by NF-κB, it might also explain the opposite outcome effects of MLN4924 *versus* CSN5i-3 on microglial inflammatory signaling, endothelial barrier integrity, and neuronal survival upon OGD/RO stress. On the other hand, the stability of proteins related to pathways found to be regulated equally by both CRL NEDDylation state-modulating drugs, such as proteins related to RNA binding/splicing, might be controlled by SRMs prone to undergo autoubiquitination and degradation upon CSN5 inhibition by Csn5i-3 [57]. An example for this mechanistic possibility from our list of identified proteins is YTH N6-methyladenosine RNA binding protein (YTHDF2), whose degradation is controlled by Skp2, a substrate adaptor that is found auto-ubiquitinated upon CSN5i-3 treatment [57]. Similarly, the SRM for the CRL responsible for the degradation of small nuclear RNA activating complex, polypeptide 5 (Snapc5) is Fbxo22, which also was found to be unstable upon CSN5i-3 treatment conditions [57].

The influence of the CSN on basal levels of IκB-α is complex. CSN2 and CSN5 knockdown were found to increase basal IκB-α in HeLa cells, but decreased them in thymocytes and HUVECs [35, 77]. Our data in microglial cells show reduced basal IκB-α and increased p65 activation upon *Csn5* siRNA knockdown. However, knockdown and inhibition of CSN5 also reduced re-accumulation of IκB-α after its TNF-induced degradation. This is in line with knockdown results obtained in HeLa cells and HUVECs [35, 77].

Cerebral microvascular endothelial cells are a key component of the BBB and their dysfunction is associated with brain disorders including stroke [91]. We found that MLN4924 attenuated OGD/RO-elicited leakiness in an hCMEC barrier model, whereas CSN5i-3 exacerbated permeability. A previous study showed that MLN4924 increased endothelial monolayer permeability in a HUVEC model and suggested a mechanism via the cullin-3-Rbx1-KCTD10 complex regulating K63 ubiquitination of RhoB [92]. In contrast and in line with our results, studies conducted in an HMEC-1 model of LPS-induced barrier dysfunction [93] and oxLDL-induced HAoEC dysfunction [94] determined protective effects of MLN4924. Explanations for the divergent outcomes could be differences in cell specificity or the addressed CRL substrate. Our data in cell assays and ex vivo organotypic brain slice cultures show that MLN4924 blocks the inflammatory and migration response of microglia, improves endothelial barrier integrity and reduced neuronal cell death following ischemia/reperfusion challenge. Inverse outcomes were obtained by CSN5i-3. CSN5i-3 was previously studied in cancer models [57, 78, 95] and explored in computational studies [96]. Majolée and colleagues observed that CSN5i-3 reduced endothelial barrier resistance in a HUVEC model via Rho GTPase [97]. While the observed link to Rho GTPases in that study was seen in endothelial cells, it is of note that we found a marked regulatory effect of MLN4924 on microglia motility. Although we did not further study the downstream signaling pathways in our present work, one might speculate that Rho GTPases are involved in this microglia effect as well. To the best of our knowledge, our study is the first report investigating the effects of a specific inhibitor of the CSN5 deNEDDylase activity in models of neuroinflammation, cerebral endothelial cell, and ischemic brain disease.

Yu et al. studied MLN4924 in the acute phase after ischemic stroke in an in vivo mouse model and found that MLN4924 reduced infarct size and improved functional outcomes via a mechanism involving neurofibromatosis 1 (NF1) and neutrophil reduction [51]. While we did not apply an in vivo model of stroke/brain ischemia, employing cell lines, primary cell cultures and ex vivo organoids, our study goes beyond the findings of Yu et al. in several ways: (i) we studied both mechanisms of neuroinflammation and ischemia/reperfusion; (ii) we scrutinized the role of several brain cell types, i.e. microglia, cerebral endothelial cells, and neurons; (iii) the role of CSN/CSN5 and that of CRL NEDDylation was studied using MLN4924 and CSN5i-3; (iv) using both inhibitors, we obtained the first proteomic profile of cullin NEDDylation status of microglial cells; (v) we identify TNF as an inflammatory mediator in brain ischemia that is down-regulated by MLN4924.

Consistent with the suggested protective role of MLN4924 in ischemic stroke [51], our data show that MLN4924 not only reduces microglial inflammation and alters microglial morphology [98], but also reduces endothelial barrier leakage and neuronal death following ischemic stress. Inversely, neuroinflammation and ischemic damage were exacerbated by CSN5i-3. While both drugs have been suggested to have a therapeutic utility in cancer, our data in conjunction with the study by Yu et al. [51] suggest that only MLN4924, and thus NEDDylation inhibition, may be a therapeutic approach in ischemic stroke. As discussed above, the stabilization of IκB-α by MLN4924 and the resistance of the IκB-α-specifying SRM βTRCP1 to auto-ubiquitination upon CSN5 inhibition by CSN5i-3 [57], may be the underlying mechanistic explanation. In agreement with this notion, we found in a therapeutic setting of our ex vivo brain model that blockade of TNF (an up- and downstream target of the NF-κB pathway) by the neutralizing antibody infliximab led to a significant improvement in neuronal survival following OGD/RO stress. To this end, the organotypic brain slice culture platform has been ascribed advantageous characteristics, i.e. being superior over primary monolayer cultures as it mimics brain cell complexity, and affording easy access and precise experimental control in comparison to in vivo settings in whole animals [68, 99, 100].

In summary, the present study unravels the microglial proteome linking several cellular pathways to the CRL NEDDylation state and provides evidence for a broad protective effect of CSN5 activity in neuroinflammation and ischemic neuronal damage. Attenuating effects on microglial inflammatory pathways, endothelial barrier integrity loss, and neuronal death were identified. CSN5-controlled inflammatory pathways such as NF-κB and TNF could be promising therapeutic targets in ischemic brain conditions.

### Supplementary Information

Below is the link to the electronic supplementary material.

Supplementary file1 (PDF 809 KB)

Supplementary file2 (XLSX 698 KB)

## Data Availability

All data and materials as well as software application information are available in the manuscript, the supplementary information, or are available from the corresponding authors upon reasonable request. The mass spectrometry proteomics data have been deposited to the ProteomeXchange Consortium via the PRIDE partner repository with the dataset identifier PXD044650.
