# Supplementary material for: The COP9 signalosome reduces neuroinflammation and attenuates ischemic neuronal stress in organotypic brain slice culture model
Source: Cell Mol Life Sci. 2023 Aug 19;80(9):262. doi: 10.1007/s00018-023-04911-8 (PMC10439869; doi:10.1007/s00018-023-04911-8)
Supplement: Supplementary file 1 — Supplementary file1 (PDF 809 KB) [file 18_2023_4911_MOESM1_ESM.pdf]

Supplementary File

The COP9 signalosome reduces neuroinflammation and attenuates ischemic neuronal stress in organotypic brain slice model

Yuan Tian[1,*], Jelena Milic[1], Laura Sebastián Monasor[2], Rahul Chakraborty[3], Sijia Wang[1,§], Yue Yuan[1], Yaw Asare[4], Christian Behrends[3], Sabina Tahirovic[2], Jürgen Bernhagen[1,3,#]

[1]Vascular Biology, Institute for Stroke and Dementia Research (ISD), LMU Klinikum, Ludwig-Maximilians-University (LMU) Munich, 81377 Munich, Germany; [2]German Center for Neuro-degenerative Diseases (DZNE), 81377 Munich, Germany; [3]Munich Cluster for Systems Neurology (SyNergy), Medical Faculty, LMU Munich, 81377 Munich, Germany; [4]Translational Stroke Research, Institute for Stroke and Dementia Research (ISD), LMU Klinikum, LMU Munich, 81377 Munich, Germany.

[#]Correspondence:
Prof. Dr. Jürgen Bernhagen
Chair of Vascular Biology, Institute for Stroke and Dementia Research (ISD)
LMU Klinikum, Ludwig-Maximilian-University (LMU), Munich, Germany
Feodor-Lynen-Straße 17, D-81377 Munich, Germany
Tel.: +49-89-4400-46151
E-mail address: Juergen.Bernhagen@med.uni-muenchen.de

* Current address: Centre for Clinical Brain Sciences, The University of Edinburgh, UK
§ Current address: Shenzhen People's Hospital, China

Table of Contents

Supplementary Figures

Supplementary Figure 1

Supplementary Figure 2

Supplementary Figure 3

Supplementary Figure 4

Supplementary Figure 5

Supplementary Figure 6

Supplementary Figure 7

Supplementary Figure 8

Supplementary Figure 9

Supplementary Figure 10

Supplementary Tables

Supplementary Table 1

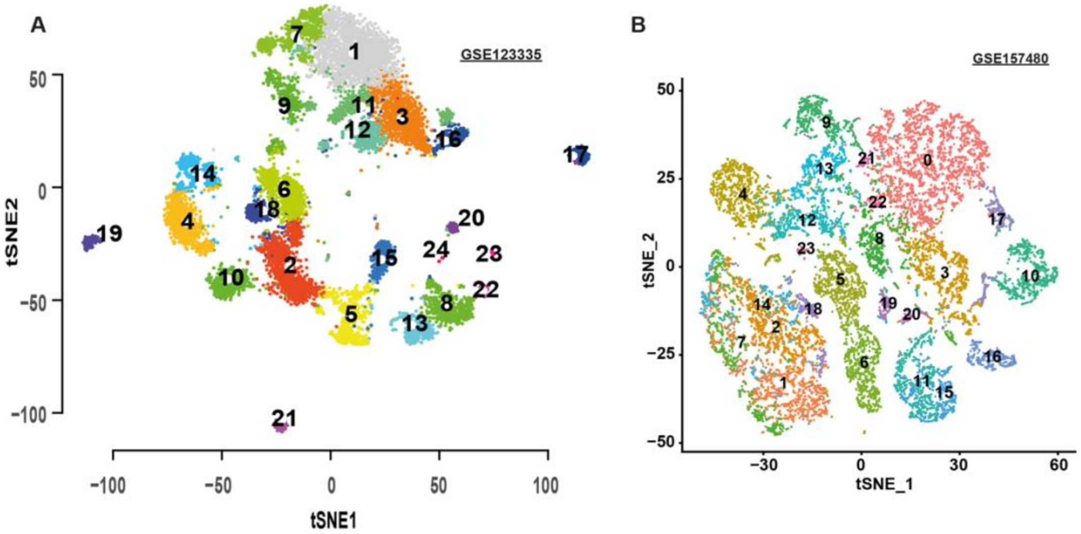

Supplementary Figure 1. tSNE plots of cell clusters from mouse neocortex and inflammatory-stimulated murine brain data sets, as used for the re-analysis of brain single cell transcriptomic data sets in the current study. (A) Single cell RNA sequencing (scRNA-seq) data from dataset GSE123335 ‘Single-cell transcriptomic analysis of mouse neocortical development’. tSNE plot shows cluster assignments of cells. Each number shows one cluster. Cluster assignments: 1, Layer II-IV; 2, INT4; 3, SVZ1; 4, INT1; 5, ganglionic eminence; 6, striatal; 7, layer II-IV; 8, astrocytes; 9, layer II-IV; 10, INT3; 11, SVZ2; 12, layer V-VI; 13, astrocytes; 14, INT2; 15, Oligodendrocytes; 16, neurons; 17, endothelial cells; 18, striatal; 19, layer I; 20, endothelial cells; 21, microglia; 22, layer V-VI; 23, choroid plexus; 24, endothelial cells. (**B**) ScRNA-seq data from dataset GSE157480 ‘IL-10 prevents pathological microglia hyperactivation following peripheral endotoxin challenge’. The tSNE plot identifies immune cell populations via cluster assignments of cells, with a focus on changes upon endotoxin stimulation. Cluster assignments: 0, monocytes; 1, T cells; 2, T cells; 3, monocytes; 4, macrophages; 5, microglia; 6, microglia; 7, NK cells; 8, monocytes; 9, dendritic cells; 10, neutrophils; 11, B cells; 12, dendritic cells; 13, macrophages; 14, T cells; 15, B cells; 16, B cells; 17, neutrophils; 18, type 2 innate lymphoid cells; 19, dendritic cells; 22, macrophages; 23, macrophages.

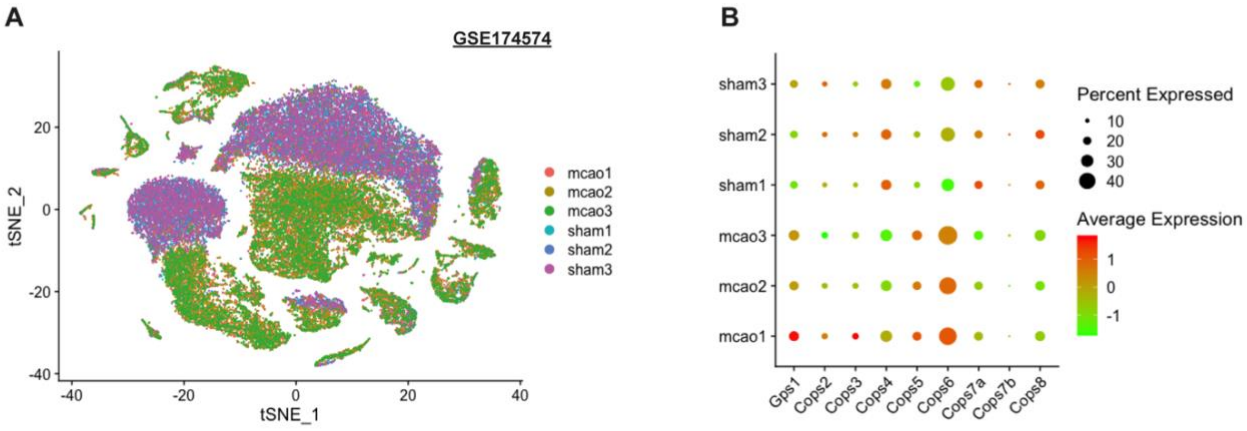

Supplementary Figure 2. Re-analysis of scRNAseq data shows that Csn subunits are upregulated upon MCAO challenge. Re-analysis of scRNA data from RNA-seq dataset GSE174574 'Single-cell RNA-seq reveals the transcriptional landscape in ischemic stroke' for COP9 subunits *Cops1-8*. (**A**) tSNE plot showing cluster assignments of cells. Each color shows one individual mouse. Mcao, mouse with middle cerebral artery occlusion (MCAO); sham, sham-operated mouse. (**B**) Dot plot visualizing the expression levels of COP9 subunits *Cops1-8* and the percentage of cells within each mouse (average expression). *Note*: most COP9 signalosome subunits, including Cops5, but most notably Cops 6, show an upregulation upon MCAO challenge.

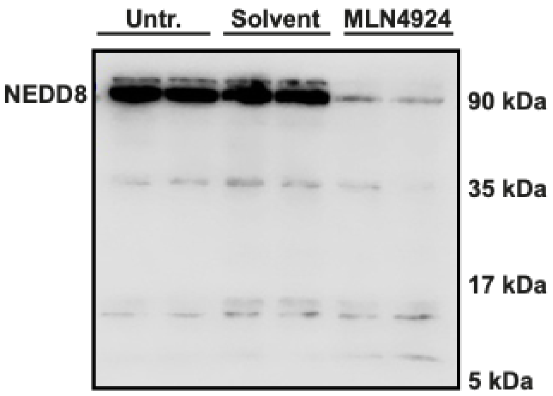

Supplementary Figure 3. Effect of MLN4924 on NEDD8 conjugation in unchallenged BV2 microglial cells. Cultured BV2 cells were treated with MLN4924 (500 nM) *versus* solvent (0.1% DMSO) for 4 h before cell lysates were prepared for Western blot analysis. Untreated BV2 cells were examined for comparison. The blot was developed with an anti-NEDD8 antibody. Depiction of the full-size Western blot. The bands showing NEDDylation at around 90 kDa coincide with bands for NEDDylated cullins (see **Figures 2A** and **6A** in the main manuscript file for comparison).

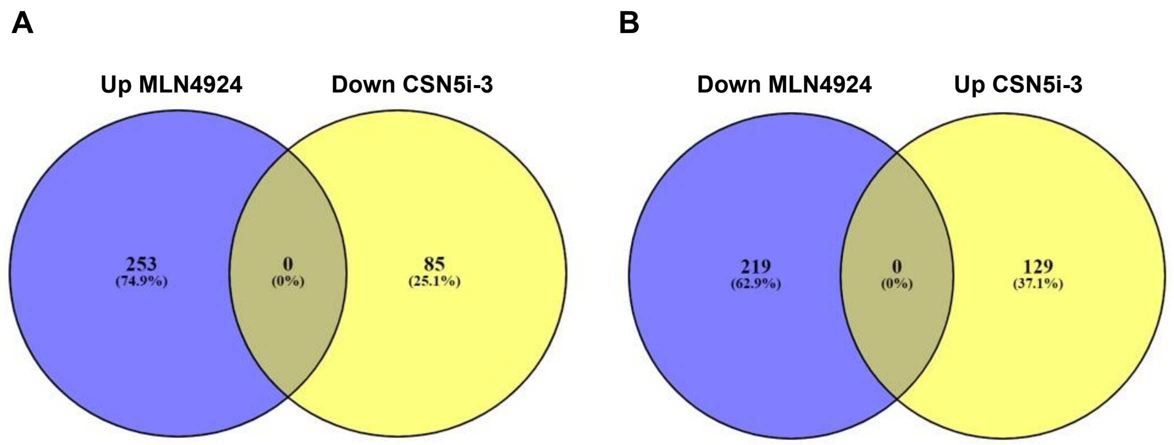

Supplementary Figure 4. Proteomic analysis shows no overlapping differentially expressed proteins when comparing opposite effects of MLN4924 and CSN5i-3 in BV2 cells. BV2 cells under basal conditions of culture stress were left untreated or were treated with control solvent (0.1% DMSO, termed solvent), MLN4924 (500 nM), or CSN5i-3 (1 μM) for 6 h before cellular proteins were prepared for proteomic analysis. Venn diagram showing overlapping proteins between the MLN4924- and CSN5i-3-treated groups. (**A)** Overlaps between proteins upregulated upon MLN4924 and down-regulated upon CSN5i-3; (**B)** Overlaps between proteins down-regulated upon MLN4924 and upregulated upon CSN5i-3. No shared oppositely regulated proteins were found in either comparison.

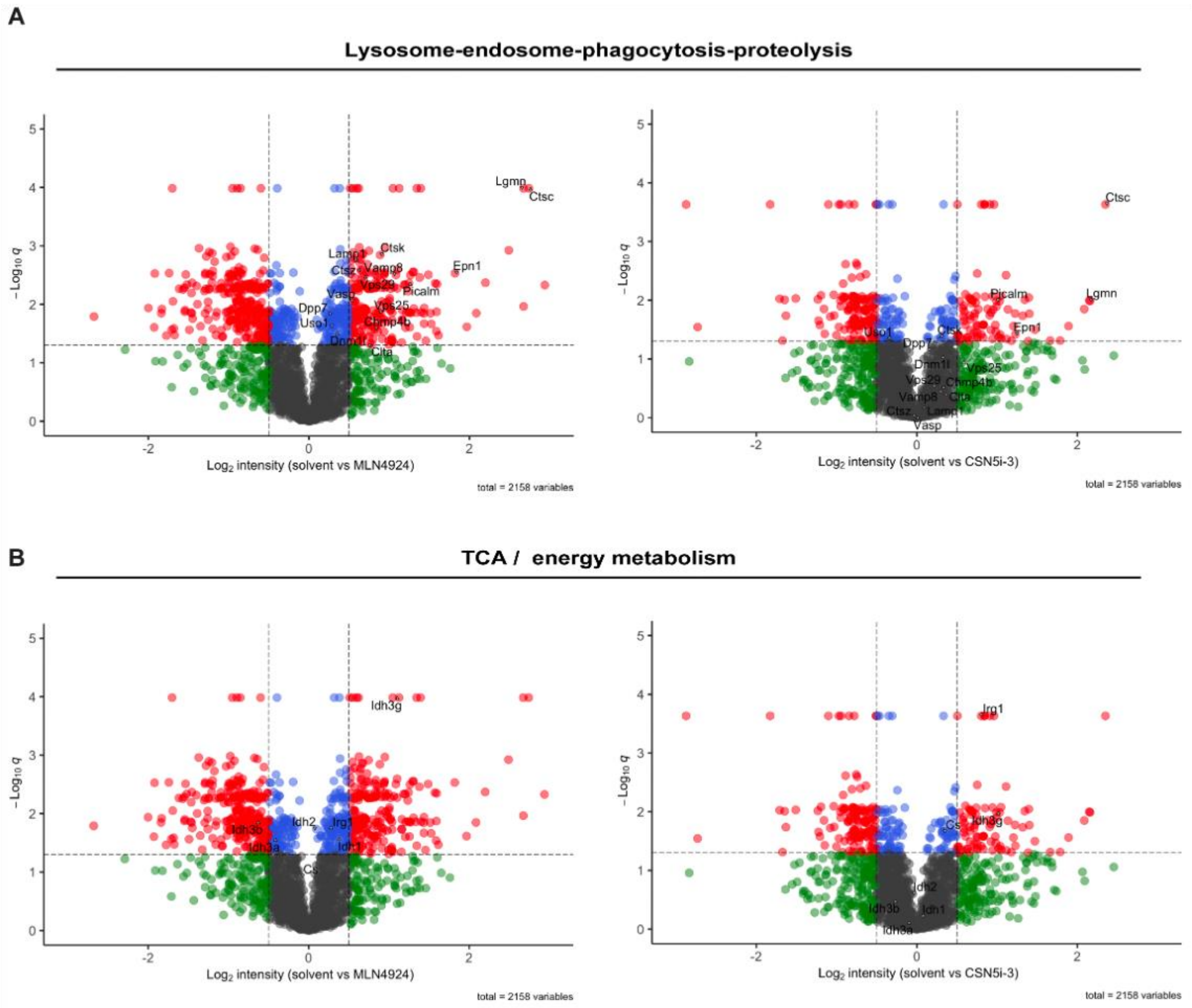

Supplementary Figure 5. Proteomic profiling of differentially expressed proteins in MLN4924 and CSN5i-3-treated BV2 cells. Comparison between solvent (0.1% DMSO)- and drug-treated conditions with a focus on proteins related to lysosomal-endosomal-phagocytosis pathways (**A**, solvent versus MLN4924 (left) and solvent versus CSN5i-3 (right)) and TCA / energy metabolism (**B**, solvent versus MLN4924 (left) and solvent versus CSN5i-3 (right)). The $\log_{10}$ student's t-test q value of each protein is plotted against the $\log_2$ intensity. The dashed horizontal line shows the cut-off of q value < 0.05, and the vertical dashed lines indicates $\log_2$ intensity > 0.5. Red data points represent proteins exhibiting Log_2 change > 1 and Log_{10} q < 0.05 significance between groups; blue data points represent proteins exhibiting Log_2 change = 0-1 and Log10 q < 0.05 significance between groups; green data points represent proteins exhibiting Log_2 change > 1 and Log_{10} q > 0.05 significance between groups. Total number of variables depicted: 2158.

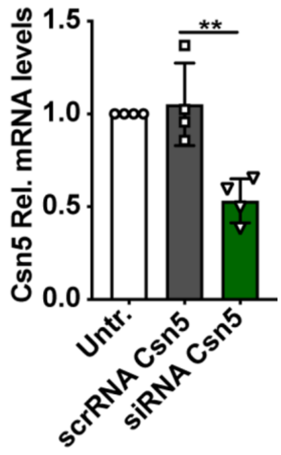

Supplementary Figure 6. CSN5 silencing leads to a reduction in *Csn5* mRNA levels in microglial cells. CSN5 silencing by siPOOL technology in BV2 microglia. Cells were transfected either with siPOOL, which targets CSN5 (siRNA *Csn5*) or scrambled control (scrRNA *Csn5*) for 72 h; untr., untreated. Relative mRNA levels of *Csn5* in BV2 cells with or without siPOOL-based knockdown are shown (mean, n = 4, ***P*<0.01, one-way ANOVA with Dunnett's multiple comparison).

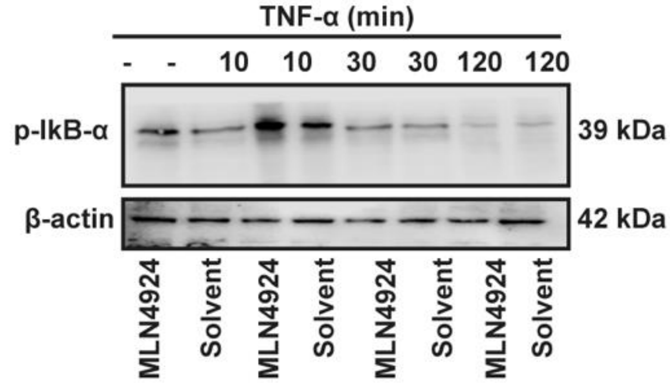

Supplementary Figure 7. Biological replicate of Figure 6E. Immunodetection of p-IκB-α and β-actin in cell lysates of hCMEC/D3 cells under TNF stimulation. See **Figure 6E** of the main manuscript for comparison.

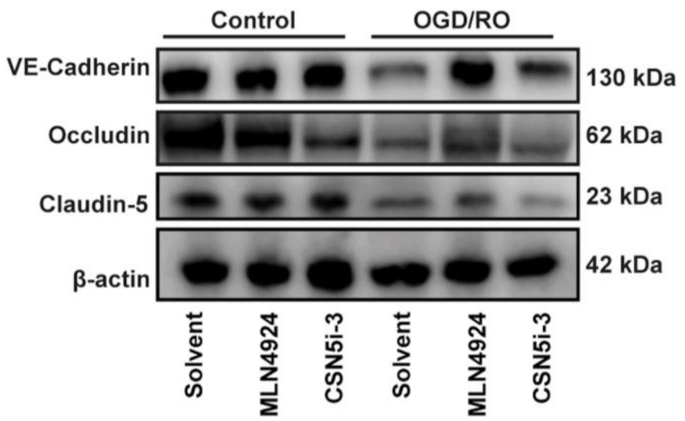

Supplementary Figure 8. MLN4924 preserves and CSN5i-3 impairs tight junction integrity of OGD/RO-stressed hCMECs. Western blot of hCMEC cell lysates developed for VE-cadherin, occludin, and claudin-5. β-actin was used as loading control. Cells were pre-treated with solvent, MLN4924, or CSN5i-3 and challenged by OGD/RO stress versus control conditions. The blot shown is representative of three independent experiments.

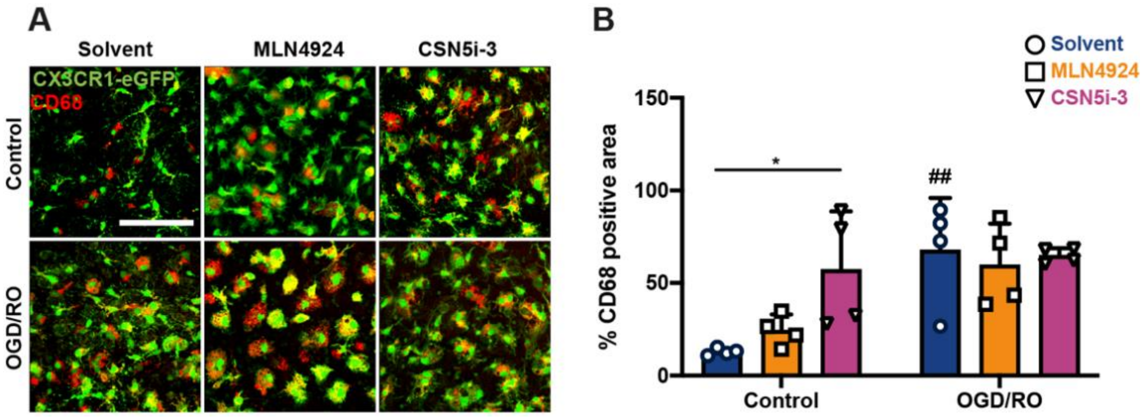

Supplementary Figure 9. Influence of CRL NEDDylation state-modifying drugs on CD68 positivity of Cx3cr1+ microglial cells. (**A**) CD68 (red) positivity of brain organ slices treated with control solvent (0.1% DMSO), MLN4924, or CSN5i-3 exposed to oxygen-glucose deprivation/re-oxygenation (OGD/RO) or control treatment without OGD as indicated. Green, Cx3cr1-EGFP+ microglia; red CD68+ cells; yellow, colocalized Cx3cr1/CD68 double-positive cells. (**B)** The area of CD68-positive cells (red) is normalized to the area of Cx3cr1-EGFP+ microglia (mean ± SD, n = 4; **P*<0.05, two-way ANOVA with Dunnett post-test for comparison with solvent; $^{\#\#}$*P*<0.01, two-way ANOVA with Bonferroni post-test for comparison with non-OGD/RO-treated control in solvent, MLN4924 or CSN5i-3 pre-treated group).

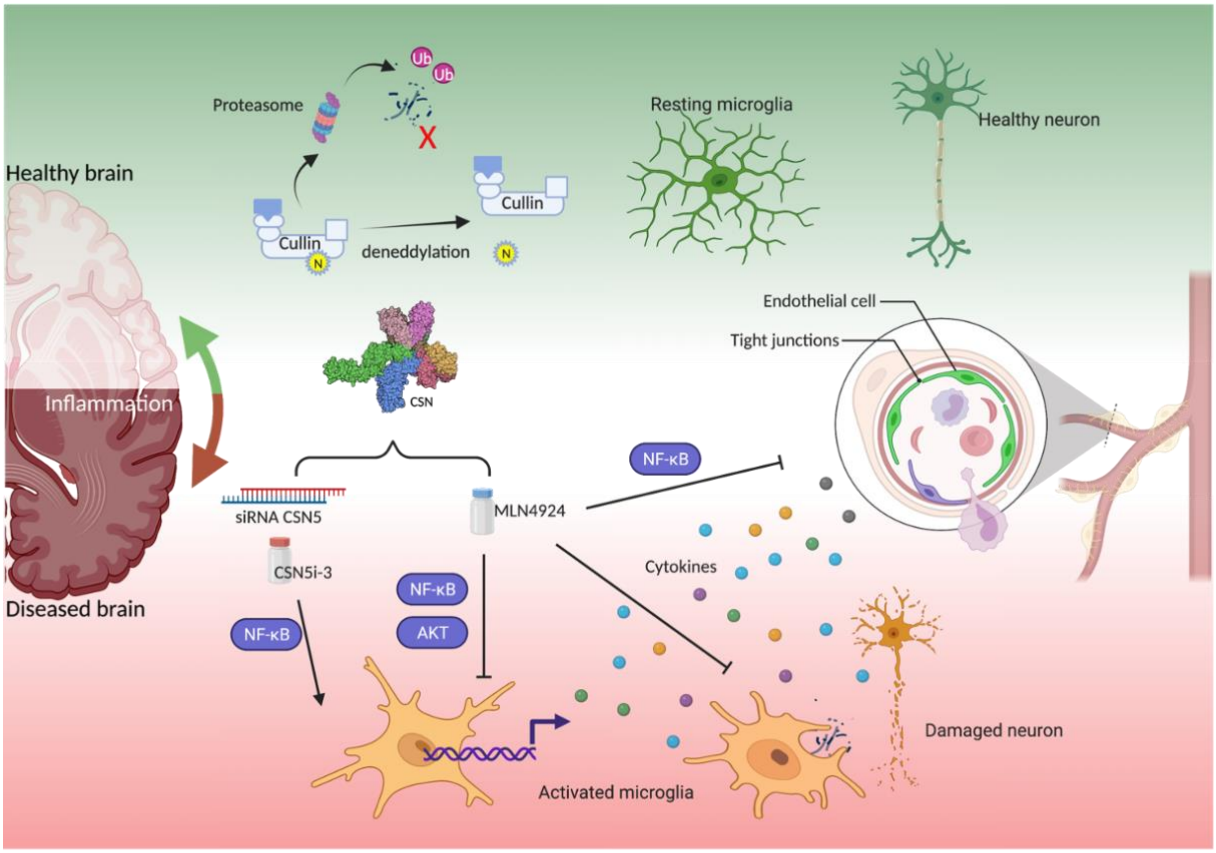

Supplementary Figure 10. Graphical summary of the role of the COP9 signalosome (CSN) and its subunit CSN5 in neuroinflammation and hypoxia-associated brain damage. Induction of brain inflammation (left) initiates microglia activation (bottom), BBB disruption and neuronal damage (right), and also leads to cytokine secretion and leukocyte infiltration (bottom, right). Based on the CRL deNEDDylation function of the CSN (top), mimicking hyperactivity of CSN5 by the NAE inhibitor MLN4924 may lead to a reduction in microglial and endothelial inflammatory signaling, BBB permeability and neuronal death. Inversely, inhibition of CSN5 activity by *Csn5* silencing or CSN5i-3 would accelerate the inflammatory processes. Our data imply the NFκB and AKT signaling pathways as key mediators. The scheme was generated with BioRender (license, ISD, LMU Munich).

Supplementary Table 1

See accompanying excel file
